# Finding the Optimal Number of Splits and Repetitions in Double Cross‐Fitting Targeted Maximum Likelihood Estimators

**DOI:** 10.1002/pst.70022

**Published:** 2025-09-11

**Authors:** Mohammad Ehsanul Karim, Momenul Haque Mondol

**Affiliations:** ^1^ School of Population and Public Health University of British Columbia Vancouver British Columbia Canada; ^2^ Centre for Advancing Health Outcomes University of British Columbia Vancouver British Columbia Canada; ^3^ Department of Statistics University of Barishal Barishal Bangladesh

**Keywords:** causal inference, cross‐fit, double robust, machine learning, sample splitting, TMLE

## Abstract

Flexible machine learning algorithms are increasingly utilized in real‐world data analyses. When integrated within double robust methods, such as the Targeted Maximum Likelihood Estimator (TMLE), complex estimators can result in significant undercoverage—an issue that is even more pronounced in singly robust methods. The Double Cross‐Fitting (DCF) procedure complements these methods by enabling the use of diverse machine learning estimators, yet optimal guidelines for the number of data splits and repetitions remain unclear. This study aims to explore the effects of varying the number of splits and repetitions in DCF on TMLE estimators through statistical simulations and a data analysis. We discuss two generalizations of DCF beyond the conventional three splits and apply a range of splits to fit the TMLE estimator, incorporating a super learner without transforming covariates. The statistical properties of these configurations are compared across two sample sizes (3000 and 5000) and two DCF generalizations (equal splits and full data use). Additionally, we conduct a real‐world analysis using data from the National Health and Nutrition Examination Survey (NHANES) 2017–18 cycle to illustrate the practical implications of varying DCF splits, focusing on the association between obesity and the risk of developing diabetes. Our simulation study reveals that five splits in DCF yield satisfactory bias, variance, and coverage across scenarios. In the real‐world application, the DCF TMLE method showed consistent risk difference estimates over a range of splits, though standard errors increased with more splits in one generalization, suggesting potential drawbacks to excessive splitting. This research underscores the importance of judicious selection of the number of splits and repetitions in DCF TMLE methods to achieve a balance between computational efficiency and accurate statistical inference. Optimal performance seems attainable with three to five splits. Among the generalizations considered, using full data for nuisance estimation offered more consistent variance estimation and is preferable for applied use. Additionally, increasing the repetitions beyond 25 did not enhance performance, providing crucial guidance for researchers employing complex machine learning algorithms in causal studies and advocating for cautious split management in DCF procedures.

**Abbreviations**
ANA: anti‐nuclear antibodies
APC: antigen‐presenting cells
ATE: average treatment effect
DCF: double cross‐fitting
DDML: double/debiased machine learning
GLM: generalized linear model
IPW: inverse probability weighting
IRF: interferon regulatory factor
MSE: mean squared error
NHANES: national health and nutrition examination survey
PS: propensity score model
RCT: randomized controlled trial
RD: risk difference
SCF: single cross‐fitting
SE: standard error
TMLE: targeted maximum likelihood estimator

## 1 Introduction

### 1.1 TMLE Framework and Cross‐Validation

Model misspecification is a challenge for epidemiological analysts working with real‐world data [1]. Double robust methods, such as Targeted Maximum Likelihood Estimator (TMLE) in combination with machine learning methods, are often touted as a potential solution [2]. TMLE is a plug‐in estimator grounded in semiparametric theory and constructed using influence functions derived from the efficient influence curve [3, 4, 5].

The use of machine learning for nuisance estimation within TMLE is commonly enabled through Super Learner, an ensemble method that combines multiple candidate algorithms. Super Learner relies on cross‐validation to evaluate and optimally weight these learners [6]. By incorporating a diverse set of candidate models, Super Learner helps address model misspecification, enhancing the robustness of TMLE in applied settings [7]. However, highly flexible learners—such as random forests or boosted trees—can violate empirical process conditions, particularly the Donsker class assumption (see Appendix A.1), which is required for valid asymptotic inference under classical theory. This has led to concerns about bias and undercoverage in confidence intervals when using such learners [8, 9, 10]. To avoid these issues, one option is to restrict the Super Learner library to simpler, Donsker‐compliant algorithms (e.g., regression splines), provided that the resulting nuisance estimators still converge fast enough to satisfy the condition that the product of their errors remains $O\left(n^{-1/2}\right)$ [10]. However, this restriction may reduce adaptivity and limit the ability to capture complex relationships in the data.

The need to avoid Donsker class conditions was anticipated in foundational work by Newey and Robins, who showed that sample splitting and undersmoothing can mitigate these issues and improve estimation in nonparametric and high‐dimensional settings [11, 12]. Building on this insight, cross‐validated TMLE (CV‐TMLE) was developed to apply TMLE within each fold of a cross‐validation scheme and aggregate results, thereby enabling the use of flexible machine learning methods without relying on Donsker assumptions [13, 14]. Despite these theoretical advantages, CV‐TMLE is not the default in the “tmle” “R”package and is less commonly used in practice, primarily due to added computational complexity. Instead, implementations in that package adopt a simpler default approach that cross‐validates only the initial outcome model, rather than the full TMLE procedure, and this approach has been shown to offer similar performance in practice [15, 16, 17].

### 1.2 Single Cross‐Fitting in DDML Framework

Recently, the Double/Debiased Machine Learning (DDML) framework has been proposed to estimate causal effects [18]. Similar to TMLE, this framework is also grounded in statistical semi‐parametric theory and aims to reduce bias when incorporating machine learning for nuisance function estimation. TMLE can be viewed as a related approach within this broader class of orthogonalized, doubly robust estimators [19, 20]. To address the risk of invalid inference from using flexible learners that violate Donsker conditions within the DDML Framework, researchers implement a single cross‐fit (SCF) procedure, dividing the available data into two parts (i.e., folds or splits). The first split is used to train (or fit) the nuisance models, such as the propensity score model and the outcome regression model. Once these models are trained on the first split, they are then used to make predictions on the second split [9]. Next, the roles of the splits are reversed, so that the previously held‐out observations now serve as the training set, ensuring that all observations receive out‐of‐sample predictions. The concept of SCF can be naturally expanded by dividing the data into multiple (p) folds or splits and by repeating the fold creation process r times, similar to a repeated p‐fold cross‐validation process with r repetitions. This repetition helps to mitigate the influence of any peculiarities in a single data partition on the final estimate, making the process more robust against potential sources of bias.

Researchers, however, note that results from the SCF procedure can be dependent on the chosen random number seed, and suggested using a higher number of splits to avoid such dependency [21]. In one application of augmented inverse probability weighting (AIPW), another double robust approach, authors have used 10 SCF splits [21]. In the DDML framework, it is recommended that p=4 or 5 splits perform better for SCF than any smaller number of splits, and p=5 splits can serve as a reasonable baseline [18, 22].

### 1.3 Adaption of Single Cross‐Fitting in TMLE Framework

Owing to its potential [23], cross‐fitting is also considered in recent TMLE implementations through “tmle3”package [24]. A recent simulation study in high‐dimensional confounding settings found that while various doubly robust methods (e.g., AIPW and TMLE) performed similarly, TMLE exhibited greater stability, and cross‐fitting was particularly beneficial for improving standard error estimation rather than point estimates, reinforcing the importance of sample splitting and careful learner selection in ensemble methods [25]. However, when additional structural assumptions, such as Hölder smoothness of nuisance functions (see Appendix A.2), are plausible, SCF may no longer be rate‐optimal, motivating extensions such as double cross‐fitting (DCF) or higher‐order estimators [12, 23].

### 1.4 Extension to Double Cross‐Fitting

DCF extends the concept of SCF by fitting the treatment and outcome models on independent and non‐overlapping data splits [26]. This separation helps mitigate overfitting and stabilize bias, particularly in high‐dimensional or complex settings. Theoretical results show that DCF‐based estimators can outperform SCF‐based ones, especially when flexible machine learning methods are used for nuisance estimation [26]. DCF has been shown to produce estimates with improved bias properties and more accurate confidence intervals. In related nonparametric estimation problems (e.g., entropy or quadratic functionals), combining sample splitting or cross‐fitting with undersmoothing has also led to near‐optimal rates under structural assumptions such as Hölder smoothness [11, 27, 28, 29].

### 1.5 Gap in the Literature

Choice of number of splits p in this procedure offers a trade‐off between reducing bias and maintaining sufficient data within each split for accurate model training. However, only a limited number of studies have been conducted within the DCF context [9, 30], and these have only used p=3. The transferability of suggestions about the optimal number of splits from the DDML (p=5) [18, 22] or single cross‐fit (p=10) literature to the DCF literature is not yet clear [21].

There is also no clear guideline in the literature about how many repetitions are necessary for DCF. While some researchers suggest that a lower number of repetitions (r=5) provides stable estimates [30], others recommend using much higher repetitions (r=100) to obtain more stable results [9, 18]. Although choosing a higher number of repetitions is associated with significantly more computational cost, we could not find any literature on how beneficial it is.

A recent Epidemiology article explained the implementation details of DCF TMLE estimators [9]. The authors used 100 repetitions and 3 sample splits (r=100, p=3) and expressed concern regarding increasing the number of sample splits. The authors argued that splitting the data too much may impair an analyst's ability to obtain reasonable results due to the reduced amount of data in each split, which is particularly problematic when using complex machine learning algorithms. They identified this scenario as a topic for future research within the double robust methods framework.

### 1.6 Aims of this Work

Motivated by this gap, our aims in this article are twofold: (1) review and explain ways to generalize DCF beyond three splits and (2) demonstrate the implications of increasing the number of splits (p) and repetitions (r) for TMLE estimators through statistical simulations. To illustrate the practical application of this DCF approach and the impact of choosing different numbers of splits in real‐world analysis, we will analyze a dataset obtained from the National Health and Nutrition Examination Survey (NHANES) 2017–18 cycle.

## 2 Materials and Methods

### 2.1 Generalization of Double Cross‐Fitting

To better understand the generalization of DCF, let us start by illustrating the process with three splits and then extend it to five splits in two different ways. In these descriptions, we use ${\mathrm{ATE}}_{p}$, ${\mathrm{ATE}}_{r}$, and ATE (without any subscript) to represent split‐specific, repetition‐specific, and overall average treatment effects (three layers), respectively. We also use Y, A, and L to represent binary outcomes, binary exposure status, and a list of confounders, respectively. Furthermore, we denote the potential outcomes under treated and untreated conditions as $Y^{1}$ and $Y^{0}$, respectively.

#### 2.1.1 Three Non‐Overlapping, Almost Equal‐Sized Splits

**FIGURE 1:**
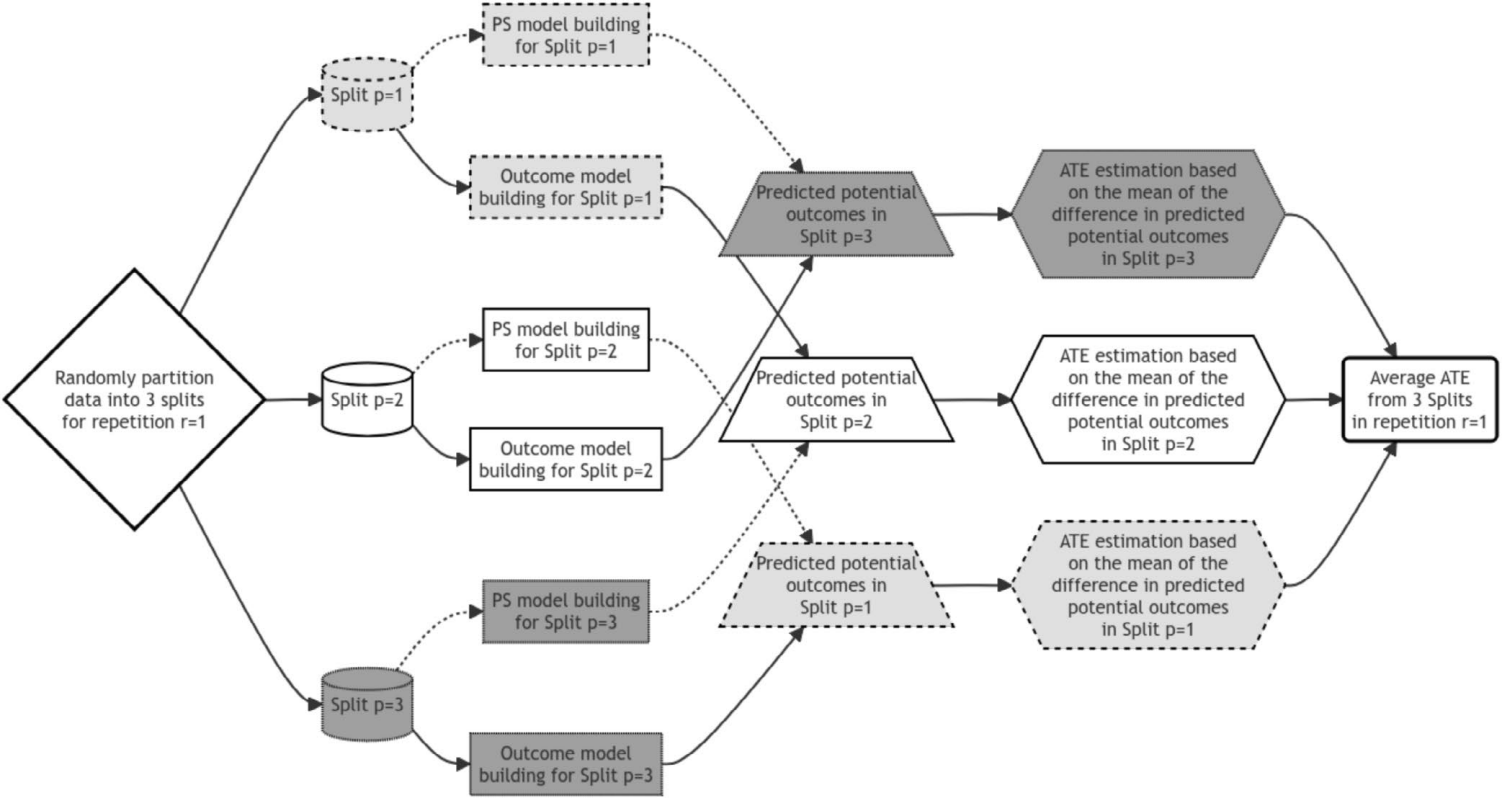
Illustration of a 3‐split double cross‐fitted Targeted Maximum Likelihood Estimator. The process is explained for obtaining a single $A\hat{T}E_{r=1}$ estimate from only one repetition. Each step using the data from the same split is color‐coded: The first split is light grey, the second split is white, and the third split is dark grey. Arrows associated with propensity score models are dotted. *Note:* ATE = average treatment effect.

For the 3‐split DCF TMLE, data is divided into three non‐overlapping, almost equal‐sized splits. Treatment and outcome models, $\hat{P}r\left[A=1|L\right]$ and $\hat{P}r\left[Y=1|A,L\right]$ respectively, are separately fitted across all 3 splits, usually through a rich set of candidate learners within the super learner framework to allow adequate flexibility to address model‐misspecification related concerns. The potential outcome predictions for each split under two different treatments are calculated using propensity score and outcome predictions from the other two discordant splits. For instance, we use the estimated propensity score model $\hat{P}r\left[A=1|L\right]$ built from split 1 and the estimated outcome model $\hat{P}r\left[Y=1|A,L\right]$ built from split 2 to predict estimated potential outcomes under both treatment conditions (${\hat{Y}}^{1}$ and ${\hat{Y}}^{0}$) in split 3. These predictions are then used to estimate the treatment effect for the third split ($A\hat{T}E_{p=3}$) based on the mean of the difference in the estimated potential outcomes. Similarly, $A\hat{T}E_{p=1}$ and $A\hat{T}E_{p=2}$ can be calculated, and the average of all three split‐specific estimates gives us the first repetition's (for r=1) treatment effect estimate (see Figure 1):

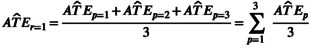




The variance of $A\hat{T}E_{r=1}$ is estimated through averaging the three variances found from three different splits:

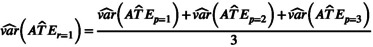




Similar to repeated cross‐validation, the treatment effect estimation is assumed to be improved by repeating the splitting process a large number of times (r). We followed the recommendation of using r=100 repetitions to obtain more stable results [9, 18]. The same process as shown in Figure 1 is then repeated r=100 times (starting from randomly partitioning the data into 3 splits), and the overall $A\hat{T}E$ is estimated based on the median of all r=100 $A\hat{T}E_{r}$ estimates from the repetitions: $A\hat{T}E_{r=1},A\hat{T}E_{r=2},\ldots ,A\hat{T}E_{r=100}$ [9, 18]. The variance of the overall $A\hat{T}E$ is calculated from the median of within $\left[\hat{\mathrm{var}}\left(A\hat{T}E_{r}\right)\right]$ and between $\left[{\left(A\hat{T}E_{r}-A\hat{T}E\right)}^{2}\right]$ variations in $\hat{A}{\mathrm{TE}}_{r}$s with respect to the repetitions r:

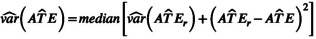




Since the DCF process requires a large number of repetitions, computing time is typically high. If some of the learners are time‐consuming to fit, the estimation process can become tedious.

As we will discuss later in the real‐world application section, it is possible that we may fail to obtain results from certain repetitions. Failures in some repetitions likely stemmed from the lack of variability in certain binary covariates within individual splits, which can cause the super learner to fail during model fitting. This issue is particularly relevant when working with datasets containing many sparse binary variables. Given that we rely on medians, even if we are missing results from a few repetitions, we can still calculate a reasonable overall ATE and corresponding variance estimates. Alternatively, researchers can choose different randomization seeds until they obtain results from all or most repetitions.

#### 2.1.2 Generalization 1: 5 Non‐Overlapping, Almost Equal‐Sized Splits (Equal Splits)

**FIGURE 2:**
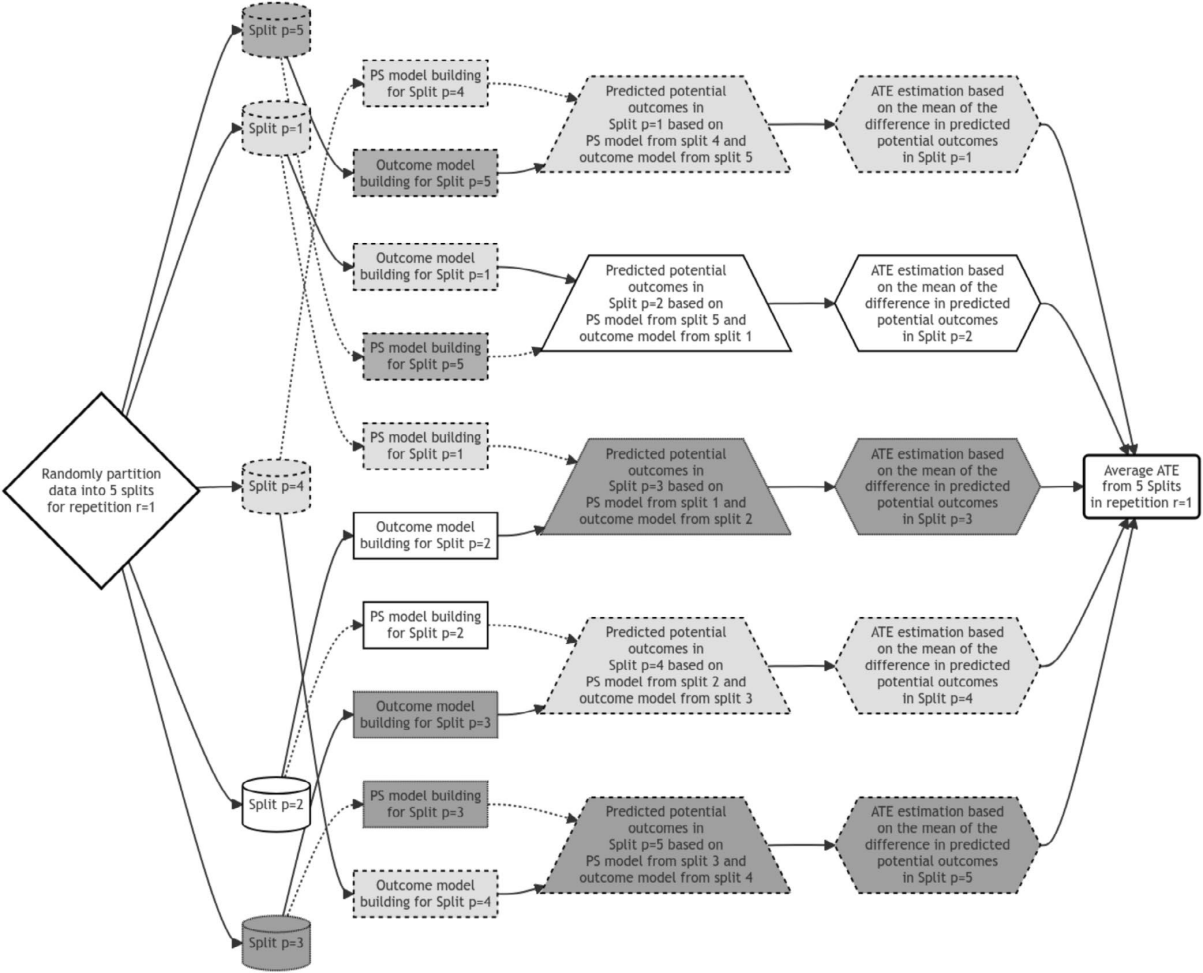
An illustration of a 5‐split double cross‐fitted Targeted Maximum Likelihood Estimator under Generalization 1. In this scenario, three discordant splits are used: One for estimating the propensity score model, another for estimating the outcome model, and a distinct split for estimating potential outcomes to obtain the treatment effect estimate. We can use super learner algorithm with cross‐validation for treatment and outcome model estimation. Each step using data from the same split is color‐coded (white or different shades of grey), and arrows associated with propensity score models are represented as dotted lines. *Note:* PS = Propensity Score Model; ATE = Average Treatment Effect.

For five splits, we can similarly extend the model fittings and obtain five $A\hat{T}E_{p}$ estimates. For instance, the propensity score model fitted using the data from split 2 and the outcome model fitted using the data from split 1 are used to predict potential outcomes under both treatment conditions in split 3, which is then used to calculate the treatment effect for the third split ($A\hat{T}E_{p=3}$). However, this straightforward generalization results in a loss of data in the calculation of $A\hat{T}E_{p=3}$, as data from the fourth and fifth splits are not used in this calculation (see Figure 2).

Although each $A\hat{T}E_{p}$ in this generalization is calculated with less data, all splits are ultimately used once in the repeated (usually sequential) process. We subsequently obtain $A\hat{T}E_{r=1}$ by averaging all 5 of the estimates obtained from each split: $A\hat{T}E_{p=1}$, $A\hat{T}E_{p=2}$, …, $A\hat{T}E_{p=5}$. Therefore,

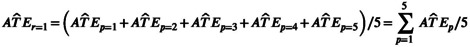




We repeat the process r=100 times to obtain the overall $A\hat{T}E$ by finding the median of all such $A\hat{T}E_{r}$s, and variance is calculated as described earlier.

The loss of data increases with a higher number of splits, such as p=10, where only 3 non‐overlapping splits (say, p=1, 2, 3) will be used to estimate, for example, $A\hat{T}E_{p=1}$ (based on propensity score model predictions from split p=2 and outcome predictions from split p=3), while data from the other 7 splits (p=4,…,10) will remain unused.

#### 2.1.3 Generalization 2: 5 Non‐Overlapping Splits with Utilizing Full Data (Full Data Use)

To fully utilize the data in calculating each $A\hat{T}E_{p}$ within each repetition, we can employ a weighted approach, where we give more emphasis in the estimation of the propensity score and outcome models. Here, one split is used for treatment effect estimation (e.g., from potential outcome predictions under two different treatments), while the remaining sample is evenly divided to develop both propensity score and outcome models. Compared to Generalization 1, this approach improves the stability of nuisance parameter estimation because a larger sample is used to fit each model. This is especially important when using complex machine learning algorithms, which benefit from larger training sets to avoid overfitting and instability [31].

**FIGURE 3:**
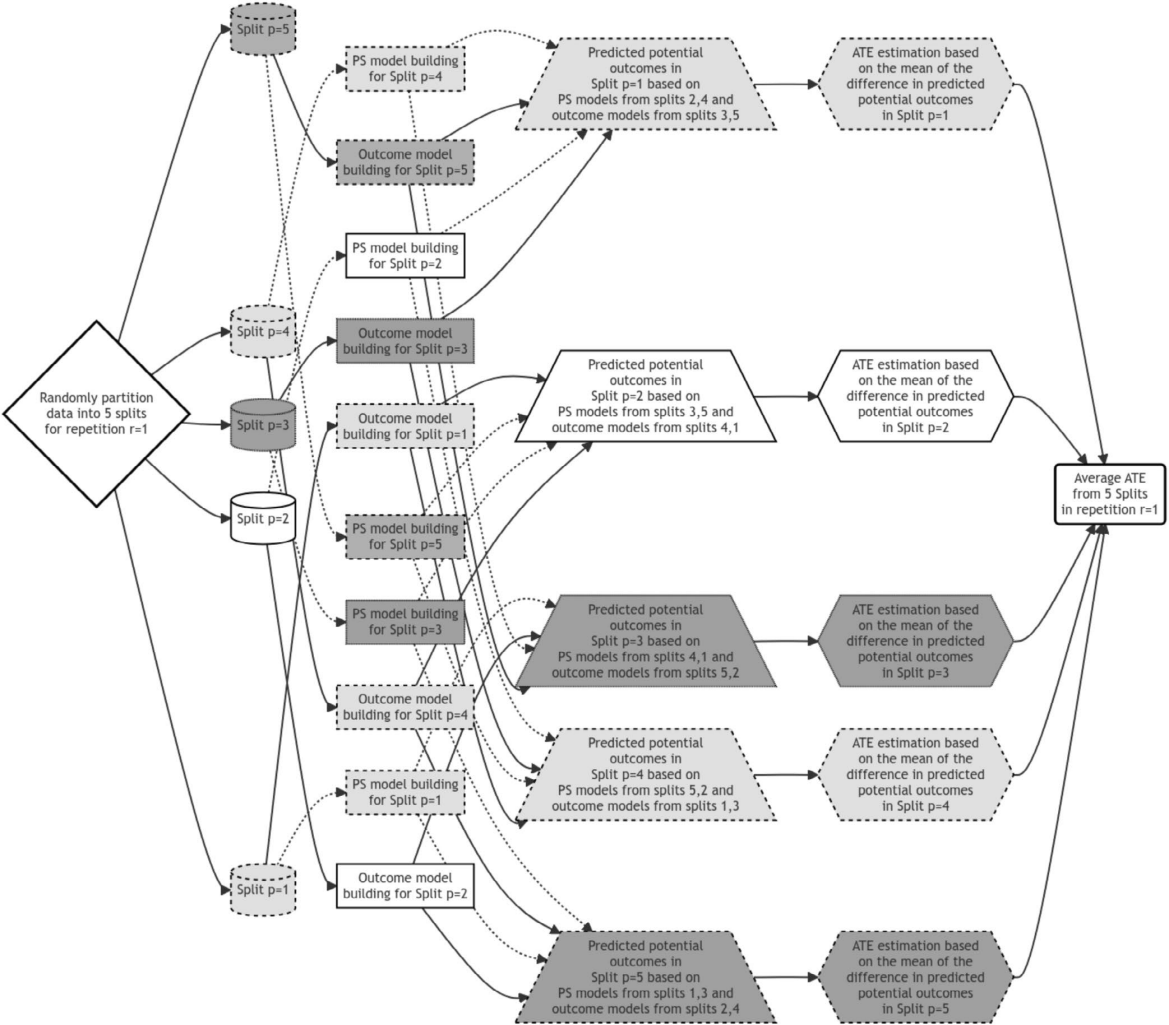
Illustration of a 5‐split double cross‐fitted Targeted Maximum Likelihood Estimator, under Generalization 2. Here, one split is employed for estimating the potential outcomes to obtain the treatment effect estimate. From the remaining splits, treatment and outcome model estimation involves more than one (but equal number of) splits for estimating these models. We can use super learner algorithm with cross‐validation for treatment and outcome model estimation. Each step using the data from the same split is color‐coded (white or different shades of grey). Arrows associated with propensity scores (models or predictions) are dotted. *Note:* PS = Propensity Score Model; ATE = Average Treatment Effect.

In the case of five splits, a single split (e.g., split 1) is dedicated to estimating the treatment effect, while the remaining four splits are allocated as follows: two splits (e.g., splits 2 and 4) are used to construct the propensity score, and the other two splits (e.g., splits 3 and 5) are employed to build the outcome model (see Figure 3). While the final ATE is still estimated using only one split's predictions, the enhanced stability of the nuisance models often leads to better‐calibrated standard errors and more reliable confidence intervals. The repetition, overall $A\hat{T}E$ and variance calculation processes remain the same as before. Even when larger number of splits such as p=9 are implemented, only 1 split is used for potential outcome predictions under two different treatments to calculate, say, $A\hat{T}E_{p=1}$, whereas remaining 8 splits will be divided equally to estimate the propensity score and outcome models (4 splits each), and thereby utilizing the full amount of data available [21].

### 2.2 DCF TMLE Versions Under Comparison

As a base case, we considered DCF TMLE with 3 splits (with a sample size of *n* = 3000), and compare the statistical properties of these estimates with those using higher number of splits in two simulation settings (see Table 1):

1. Different Sample Sizes: DCF TMLE with *p* = 3, 5 and 10 splits when calculated under different sample sizes: *n* = 3000 (under Generalization 1) versus *n* = 5000.
2. Different Generalizations of DCF: DCF TMLE with *p* = 3, 5 and 9 splits when calculated under Generalization 1 (base case) versus 2 (both with sample size *n* = 3000). We considered DCF TMLE with 3 splits as the base case, as under both generalizations, the results will be the same for the DCF TMLE with 3 splits, allowing us to compare the results.

**TABLE 1:** Summary of scenarios considered in the simulation.

Scenario	Sample size (n)	Number of splits (p)	Generalization	Number of repetitions (r)
Base scenario	3000	3, 5, 10	1	100
Different sample size	5000	3, 5, 10	1	100
Different generalization	3000^b^	3, 5, 9^a^	2	100

*Note:* Base case scenario.

^a^ We intentionally used p=9 instead of 10 for Generalization 2, so that we can assign an equal number of splits in the treatment and outcome model fitting (e.g., 4 splits each).

^b^ We did not consider higher sample size (e.g., n more than 3000) for Generalization 2 (full data), as more data are available in this setting to estimate propensity score and outcome model estimation by design.

To understand the behavior of the DCF TMLE estimator under different number of repetitions (*r* = 1, 5, 10, …, 95, 100), we saved all the granular results under the base scenario.

#### 2.2.1 Non‐Cross‐Fit TMLE

Given the availability of theoretical results comparing single and double cross‐fitting, we are not going to compare the results from SCF vs. DCF [26]. Simulation results also exist comparing SCF with the non‐cross‐fit version of TMLE [21]. However, to allow readers to have a sense of the worst‐case scenario, a non‐cross‐fit version of TMLE has been added to our comparison.

#### 2.2.2 Super Learner Specification to Combat Model Misspecification

In all these DCF TMLE estimators, to flexibly estimate both the propensity score and outcome models, we used the same super learner algorithm with 10‐split cross‐validation [32]. The following candidate learners were used within the super learner: logistic regression, generalized additive models (with 4 and 6 splines, respectively), a neural network (2 units in the hidden layer), random forest (with 500 trees and at least 20 individuals per leaf), and empirical mean. To reflect the real‐world analysis scenario, data generating covariates were supplied as main effects in the super learner, without correctly transforming the covariates as was done in the data generation process.

### 2.3 Data Generation Mechanism

We utilized a known data‐generating mechanism as described in Zivich and Breskin (2021) [9]. Appendix B provides specific details of the data generation distributions and equations. In brief, outcome (Y), and exposure (A) variables were binary. This simulation involves generating data for the covariates (L) such as age, natural‐log transformed low‐density lipoprotein, diabetes, frailty, and risk score, along with treatment and potential outcomes. To simulate real‐world scenarios, complex and non‐linear functional forms are employed in the data generation process, to study whether data adaptive machine learning methods can recover the true effect even when we mis‐specify the covariates in the models. The treatment effect of interest (ψ) is estimated by computing the difference between a large number of potential outcomes under two distinct treatment conditions ($Y^{1}$ and $Y^{0}$, respectively).

### 2.4 Measures of Performance

We report the effect measures in terms of risk difference (RD). We used R packages Crossfit to apply the DCF procedure [33, 34] and rsimsum to calculate the measures of performance [35]. Monte Carlo iterations were conducted to simulate 2000 datasets, consistent with prior work using the same data‐generating mechanism [9].

We compared the results from these 2000 simulated datasets in terms of the following measures: (1) bias, (2) two types of standard errors (SE): average model SE (the average of estimated SEs obtained from a model over repeated samples), and empirical SE (the standard deviation of treatment effects estimated over repeated samples), (3) coverage probability of 95% confidence intervals [36].

Additionally we have also reported the following: (i) mean squared error (MSE), (ii) relative percentage in error in average model SE (measures the percentage by which the average model SE either surpasses or falls short of the empirical SE, with the latter serving as the reference point in the denominator), (iii) bias‐eliminated coverage and (iv) Zip plot.

#### 2.4.1 Rationale for Focusing on SEs

Below we present the results of our simulation studies. Our findings reveal a pattern of bias, yet the bias is negligible across different sample sizes and generalizations, indicating the efficacy of both generalizations in mitigating bias. Consequently, the probabilities of coverage and bias‐adjusted coverage are fairly comparable in each scenario. In this work, we primarily focus on the influence of the number of splits in the DCF procedure on “coverage”. Given the minimal impact of bias, our analysis focuses on the relationship between the average model SE and empirical SE, which primarily determine whether coverage probabilities are near or deviate from the nominal level. This comparison of SEs further explains the mechanism by which the choice of DCF splits influences the success or failure of these approaches in achieving nominal coverage.

## 3 Results

### 3.1 Different Sample Sizes

#### 3.1.1 Bias

In the simulation study, we compared sample sizes ranging from n=3000 to n=5000 under Generalization 1 (equal splits). As the sample size increased, bias levels reduced while retaining a similar pattern (Appendix Figure C.1). When we increased the number of splits from the base case of three (p=3) to five (p=5), we observed a decrease in bias levels. Although a slight increase in bias was observed when increasing splits to p=10, these differences were within the Monte Carlo standard error (MC SE ≈0.0004) and thus not statistically distinguishable.

#### 3.1.2 SEs and Coverage

**FIGURE 4:**
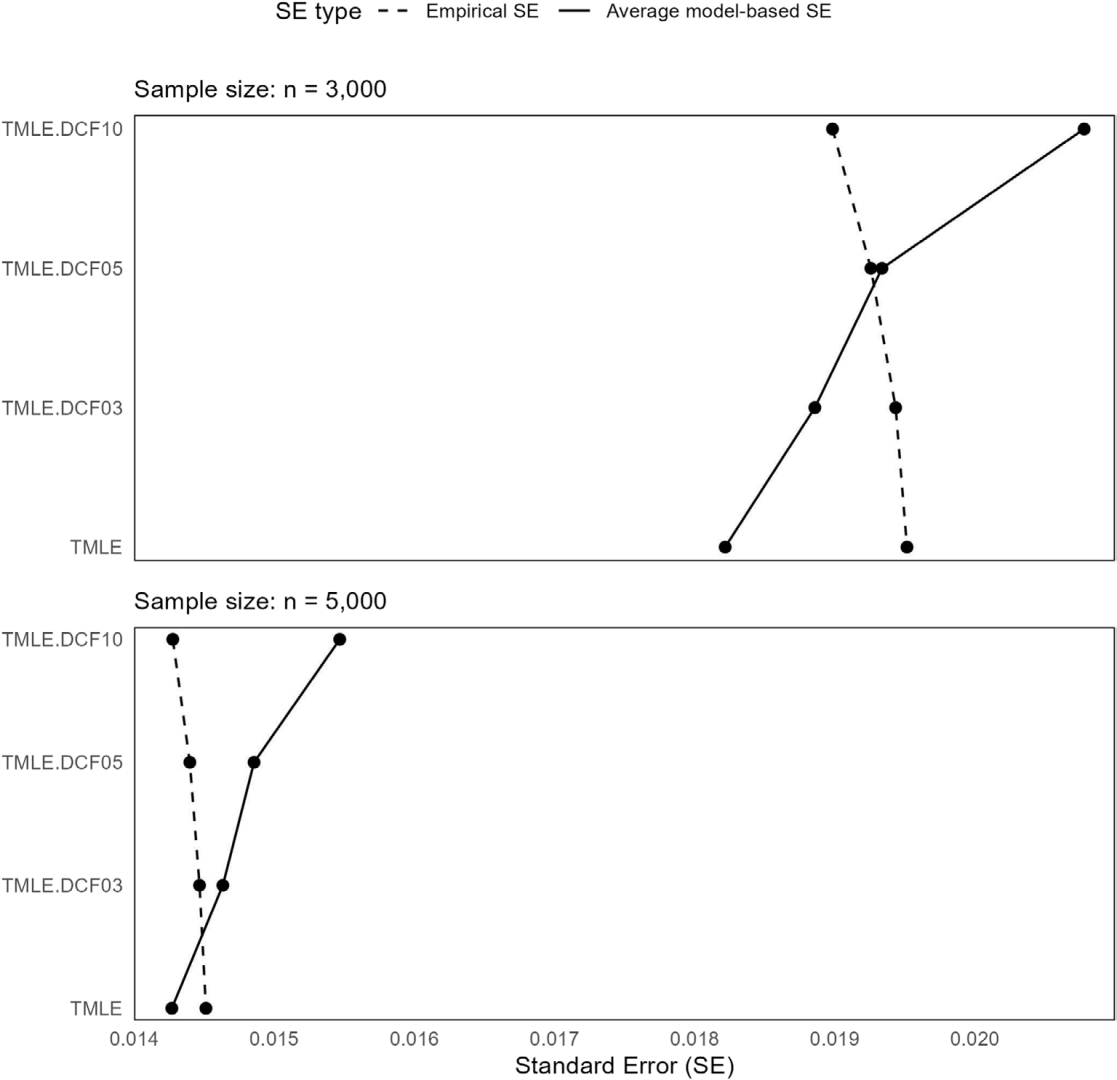
Comparing different types of standard errors from the simulation under two different sample sizes under Generalization 1. *Note:* TMLE: Targeted Maximum Likelihood Estimation; DCF: Double cross‐fitting; DCF03 to 10: DCF with p=3 to 10 splits.

The empirical SE showed a subtle decreasing trend from p=3 to p=10 and, as expected, decreased with an increase in sample size (Appendix Figure C.2). Given the minimal bias and the small differences between methods (often within the MC SE), the MSE closely mirrored the empirical SE (Appendix Figure C.3). Conversely, the average model SE increased as the number of splits increased (Appendix Figure C.4). This is expected, as a higher number of splits results in a smaller sample size in each split (see Figure 4 for a comparison of trends between two types of SEs). However, we also note that the gap between empirical and model‐based SEs does not uniformly narrow with larger sample sizes, suggesting that the model‐based SE under Generalization 1 may not consistently estimate the true sampling variability of the ATE across different split configurations. This potential inconsistency highlights a limitation of Generalization 1 when used for variance estimation.

**FIGURE 5:**
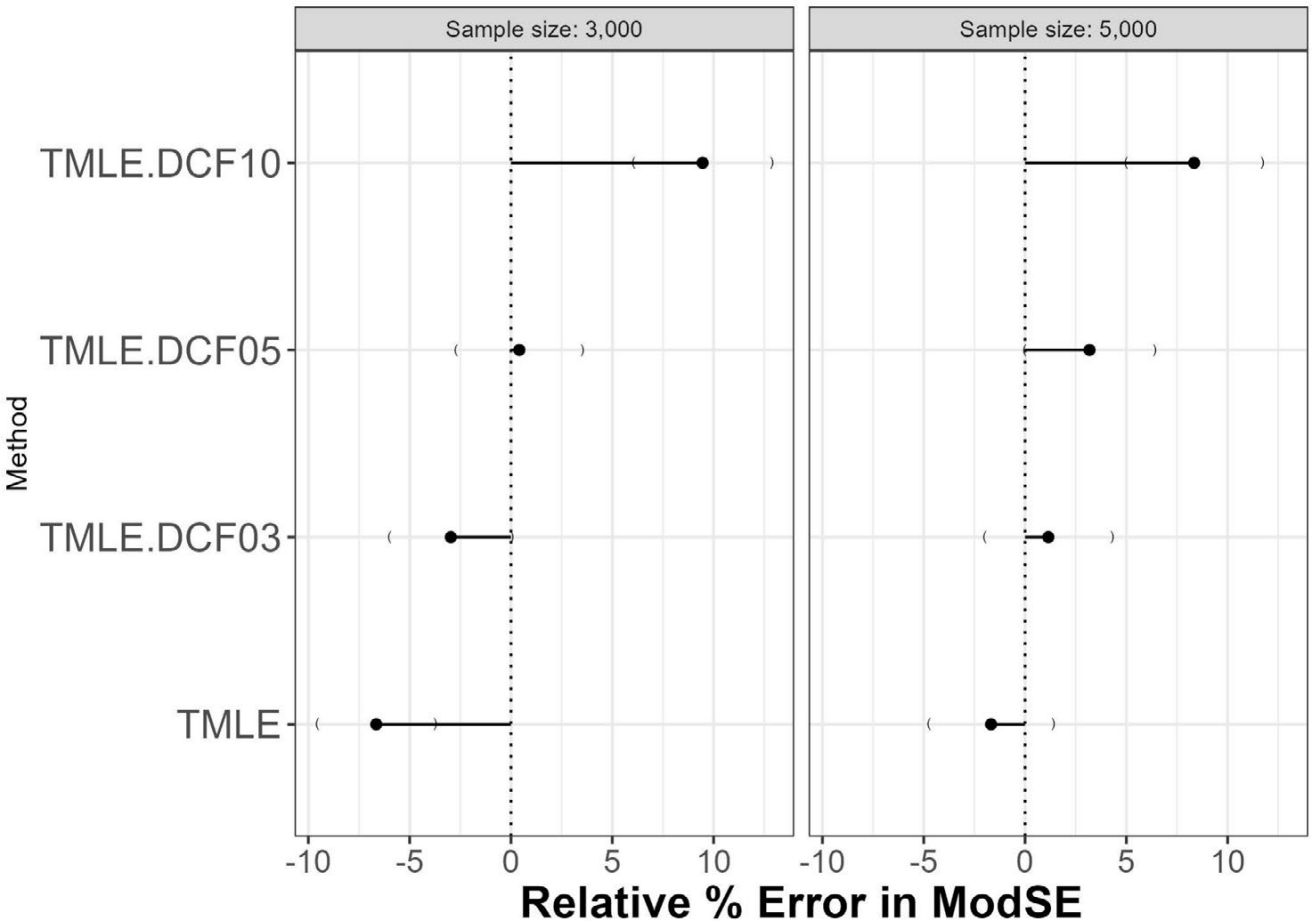
Simulation results comparing the relative errors in model standard errors under two different sample sizes under Generalization 1. *Note:* TMLE: Targeted Maximum Likelihood Estimation; DCF: Double cross‐fitting; DCF03 to 10: DCF with p=3 to 10 splits.

Since the empirical SE and average model SE follow different patterns, the relative percent error in average model SE was lowest for p=5 with n=3000. However, the error was minimal for p=3 with n=5000 (see Figure 5). The coverage and bias‐eliminated coverage plots (Appendix Figures C.5 and C.6) showed that p=5 achieved near‐nominal coverage for both sample sizes. While p=3 appeared slightly better for the larger sample size, this improvement was within the Monte Carlo error margin and may not be statistically meaningful. Corresponding Zip plots are presented in Appendix Figure C.7.

### 3.2 Different Generalizations of DCF

#### 3.2.1 Bias

We compared various statistical measures across different splitting approaches (equal splits versus full data). The full data approach (Generalization 2) showed a pattern of somewhat increased bias with a higher number of splits, but the difference of bias compared to Generalization 1 approach was negligible (Appendix Figure C.8).

#### 3.2.2 SEs and Coverage

**FIGURE 6:**
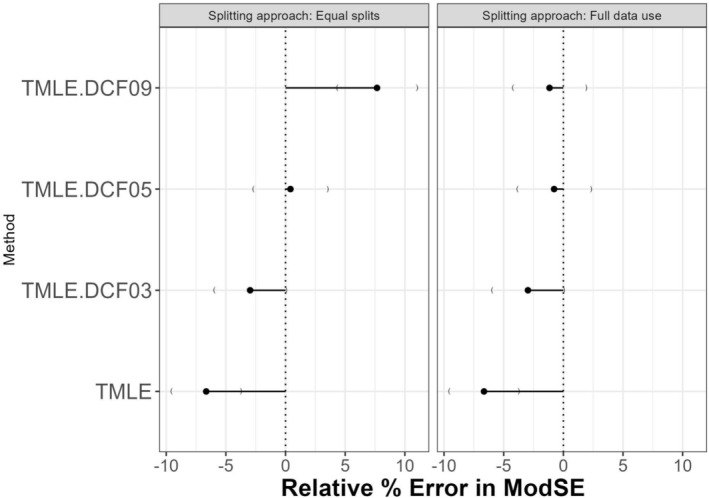
Simulation results comparing the relative errors in model standard errors under two different generalizations of double cross‐fitting for the sample size 3000. *Note:* TMLE: Targeted Maximum Likelihood Estimation; DCF: Double cross‐fitting; DCF03 to 9: DCF with p=3 to 9 splits.

Empirical SEs from all DCF methods under the full data approach were practically the same. However, model‐based SEs were slightly lower for p=3, resulting in greater underestimation of variability (i.e., larger negative relative error). Among all configurations, p=5 consistently showed the smallest relative error in SE at −0.77%, compared to −2.97% for p=3 and −1.16% for p=9, suggesting the best calibration (see Figure 6). Coverage patterns mirrored these findings, with DCF with p=5 achieving the highest bias‐adjusted coverage (94.70%), marginally outperforming DCF with p=3 and DCF with p=9 (both ≈94.35%−94.55%). These results suggest that within Generalization 2, using p=5 strikes the best balance across SE accuracy and confidence interval coverage.

Under Generalization 1, model‐based standard errors increased notably with the number of splits, and the relative percentage error in standard error rose from negative to strongly positive values (e.g., from −6.6% for DCF with p=3 to +9.5% for DCF with p=10 at n=3000; Appendix Table C.1). This suggests that the model‐based SEs increasingly overestimate the true variability as more splits are used. In contrast, Generalization 2 (which uses more data for nuisance estimation) showed more stable SE calibration, with relative SE error remaining near zero (ranging from −3% to −1.2%; Appendix Table C.3) across all splits. These results indicate that Generalization 2 may offer more consistent variance estimation across different split configurations, especially when using a moderate number of splits. Please see Appendix C for additional details on the simulation results.

### 3.3 Impact of Increasing the Number of Repetitions

**FIGURE 7:**
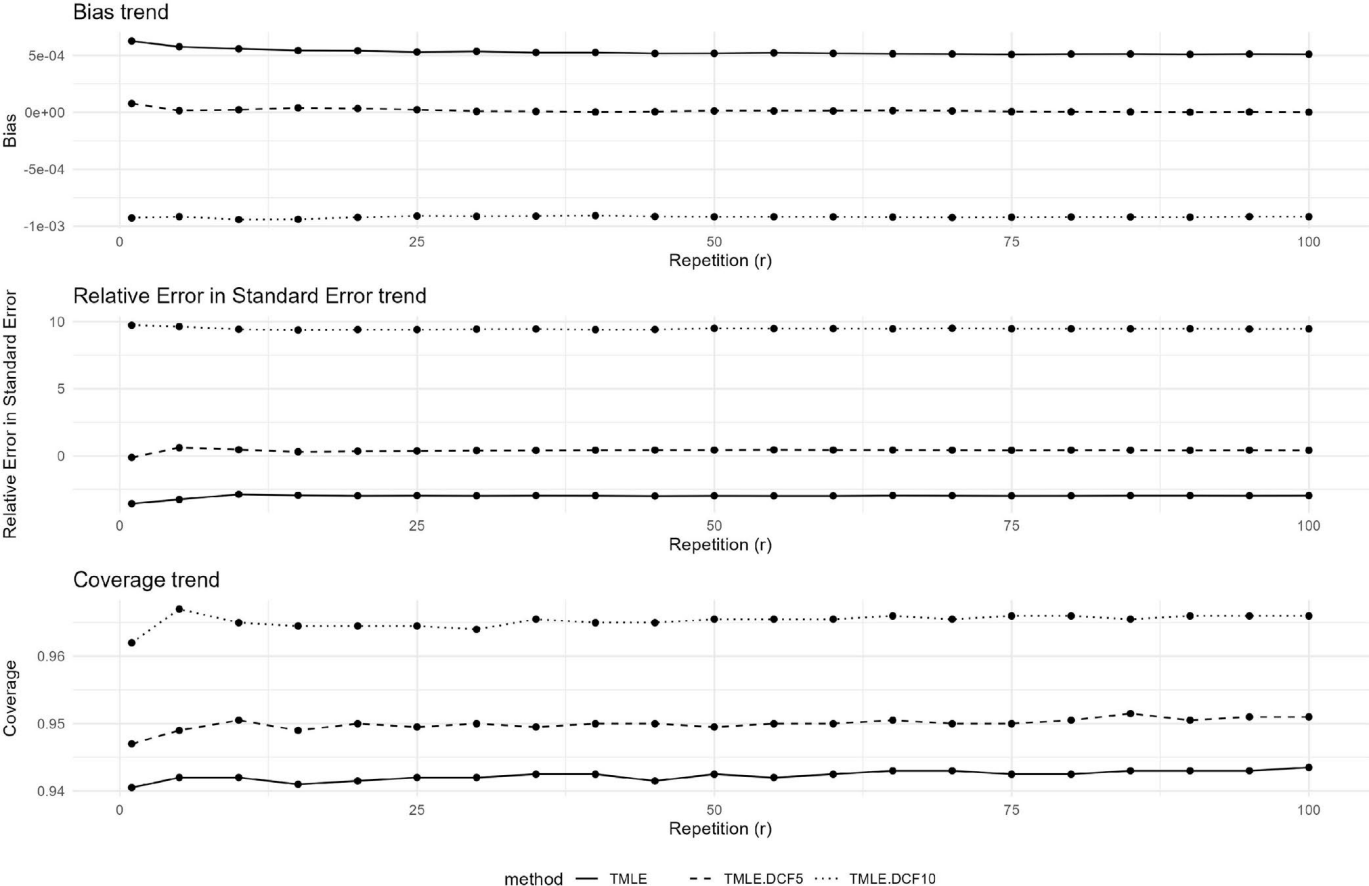
Simulation results comparing bias, relative errors in model standard errors and coverage for the double cross‐fitting TMLE under different repetitions (r = 1, 5, 10, …, 95, 100) under Generalization 1. *Note:* TMLE: Targeted Maximum Likelihood Estimation; DCF: Double cross‐fitting; DCF03 to 10: DCF with p=3 to 10 splits.

Figure 7 shows the patterns of bias, relative errors in model standard errors, and coverage for the DCF TMLE under different repetitions (r = 1, 5, 10, …, 95, 100) under the base simulation scenario (under generalization 1). The results from these estimators under r=25 repetitions versus r=100 repetitions are not notably different, with differences usually within three decimal places, except for relative errors in model standard errors (which is a ratio).

The density plots depicting the distribution of performance metrics across various analytical methods (TMLE.DCF3, TMLE.DCF5, TMLE.DCF10, all with r=100) are shown in Appendix Figure C.22. The densities are mostly non‐overlapping between the methods considered and exhibit low variability. This clearly demonstrates that the results were more sensitive to the choice of the number of splits than to the choice of the number of repetitions. As shown in that figure, the presence of outliers indicates why choosing r=1 (or a very low r) can be problematic.

## 4 Real‐World Analysis

### 4.1 Estimates Under Increasing DCF Splits

We analyzed real‐world data obtained from the NHANES 2017–18 cycle. We were interested in the association between obesity and the risk of developing diabetes from this sample. Given the observational nature of the data, there were 24 (13 categorical and 11 continuous) investigator‐specified covariates that included demographic information, health history and behavior, and clinical or laboratory measurements. On top of that, an additional 142 proxy covariates (empirical covariates) based on prescription medications were added to reduce the impact of residual confounding [37, 38]. The dataset consisted of n=2418 subjects, which is comparable to one of our simulation scenarios (n=3000). Details for reproducing the analytic NHANES data are available elsewhere [39].

Given the extensive number of covariates and proxy variables in this analysis, there is a potential risk of overfitting the results. In the simulation study, we varied the number of splits from p=3 to 10 to balance computational feasibility with statistical stability. However, given the lower computational and resource burden in a single real‐world data analysis, we extended the range to p=3 to 15 to further explore the impact of additional splits on performance of the DCF TMLE method. In constructing the super learner, we used a similar roster of candidate learners, excluding the neural network learner, to avoid non‐convergence issues when estimating both the treatment and outcome models. Similar to our simulations, we utilized r=100 repetitions in all DCF analyses, also included one non‐cross‐fit TMLE. To maintain our focus on DCF TMLE implementation and its resulting estimates, we have set aside complex survey design features, such as strata, clusters, and survey weights, to avoid additional methodological and software implementation complexities [40, 41]. The objective of this exercise is to demonstrate the impact of varying the number of DCF splits, without drawing causal, clinical, or nationally representative conclusions.

**FIGURE 8:**
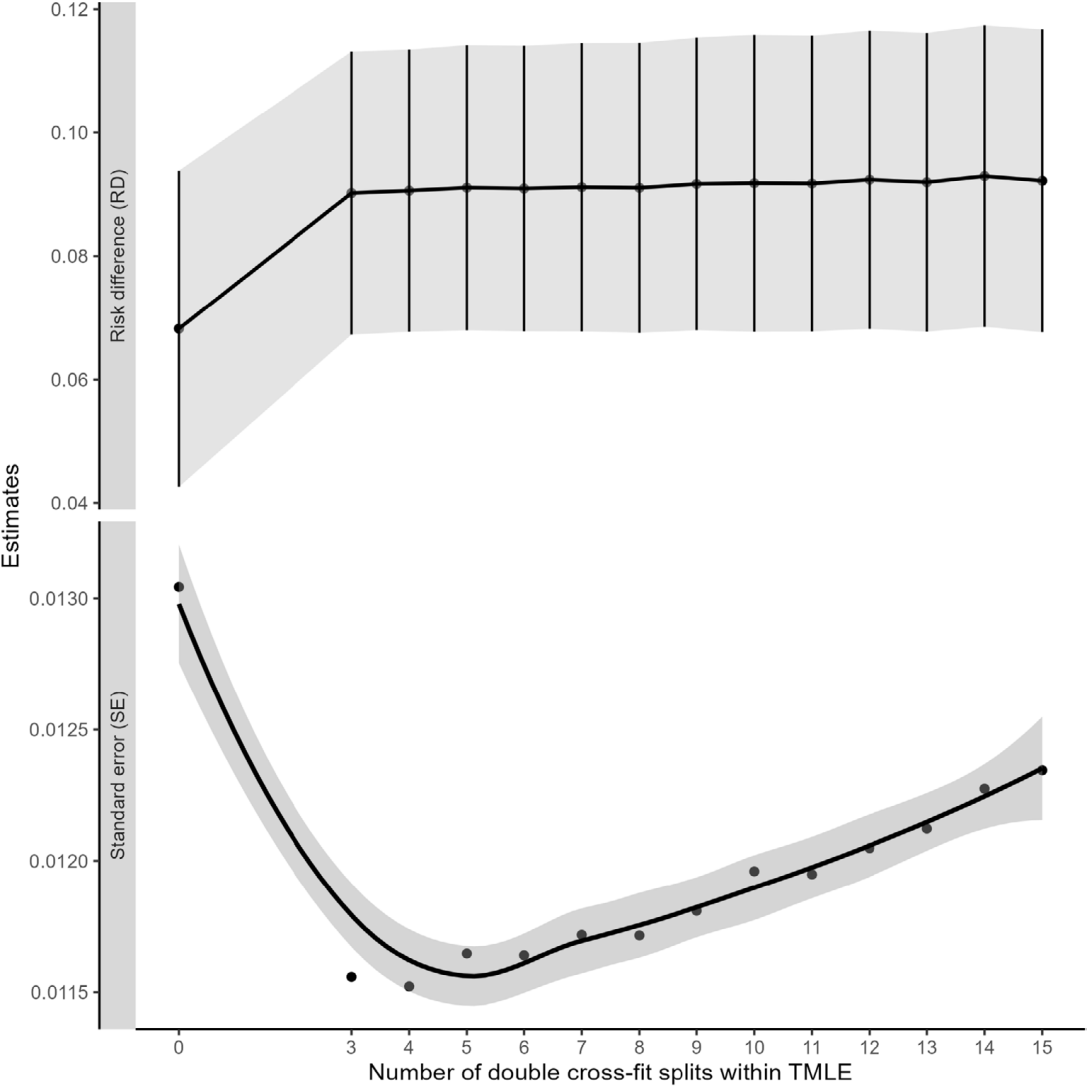
Risk difference and smoothed standard error estimates (to facilitate visualization of the trend) from the analysis of the association between obesity and the risk of developing diabetes using NHANES 2017–18 cycle data. Values plotted above zero represent the estimates obtained from a non‐cross‐fit version of TMLE, while all other values correspond to the double cross‐fitted version of TMLE associated with Generalization 1 (equal splits). *Note:* TMLE: Targeted Maximum Likelihood Estimation.

Results from Generalization 1 (equal splits) are summarized in Figure 8. The estimated RDs for DCF TMLEs from different numbers of splits were close, varying between 0.09 and 0.093. However, the model SEs exhibited an increasing trend as the number of splits increased, indicating a potential disadvantage of having too many splits. A similar pattern in the average model SE was observed in the simulation settings, as illustrated in Figure 4. For Generalization 2 (full data use), the RD estimates from the DCF estimates ranged from 0.085 to 0.091, but the SEs were relatively stable, ranging from approximately 0.0156 to 0.0158 for moderate to high numbers of DCF splits. Although the RD estimates from both generalizations remained similar, the results from the non‐cross‐fit TMLE estimates differed significantly.

### 4.2 Computing Time

We utilized a high‐performance computing platform to perform the calculations. For the NHANES data analyses, all DCF versions of TMLE were executed on a single node with 50 tasks (equivalent to using 50 CPU cores). In contrast, the non‐cross‐fitted version was run with a single core. We allocated up to 50 GB and 186 GB of memory for Generalization 1 and 2, respectively.

**FIGURE 9:**
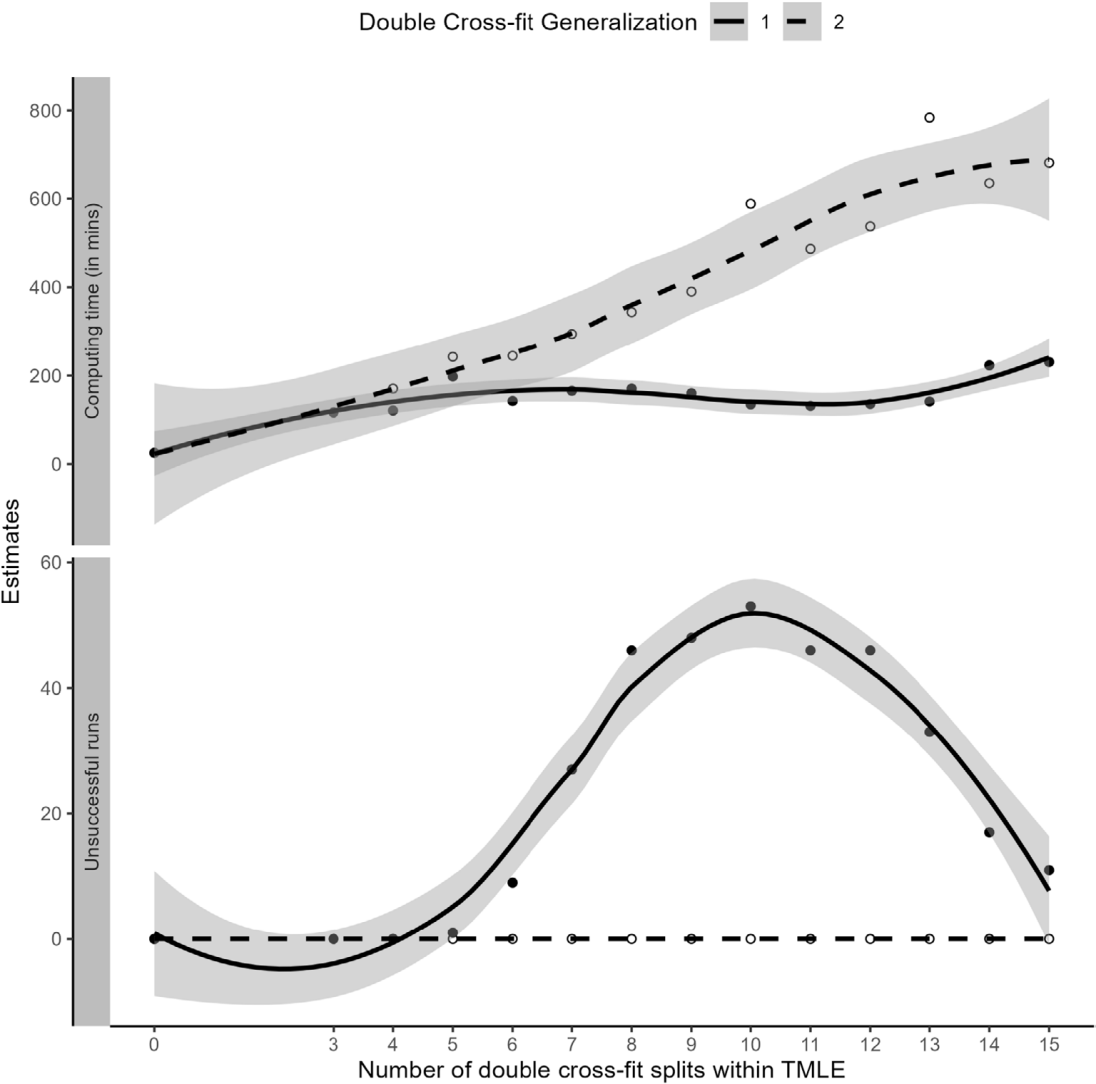
Smoothed computing time and the number of unsuccessful runs (i.e., those repetitions that failed to produce any treatment effect estimates) from the analysis of the association between obesity and the risk of developing diabetes using NHANES 2017–18 cycle data. *Note:* TMLE: Targeted Maximum Likelihood Estimation.

The computing times (in minutes) for both generalizations of DCF are presented in Figure 9. From the top panel of the figure, it is evident that the computing time for Generalization 1 (equal splits, depicted with a solid line) generally showed an increasing pattern. However, there were instances where the computing time fluctuated. We have also plotted the number of unsuccessful runs (out of r=100 repetitions), which are those repetitions that failed to produce any treatment effect estimates. In the NHANES dataset, the presence of many binary variables (e.g., 142 proxy covariates) means that with a smaller selected subset of data within a specific split, some variables might exhibit no variability (e.g., all values of those variables in the chosen split are either 0 or 1), potentially causing the corresponding super learner to fail. Given that the overall patterns of median ATEs and corresponding SEs remained relatively steady (as seen in Figure 8), we did not attempt to identify new randomized seed values to ensure the attainment of all 100 repetition‐specific ATEs. This may account for some of the variability observed in the computing time plot for Generalization 1 (Figure 9 bottom panel).

For Generalization 2, as we used an increasingly larger number of samples for the estimation of both the propensity score and outcome models, all repetition‐specific ATEs were successfully obtained (Figure 9 bottom panel, depicted with a dashed line). However, the computing time increased significantly with the increase in the number of splits (p) (Figure 9 top panel).

### 4.3 Choice of the Number of Repetitions

In our analysis, we chose to use r=100 repetitions within the DCF procedure, following suggestions from previous literature [9, 18]. However, this decision entails a significant computational burden, as the entire process needs to be repeated 100 times.

Using the 100 estimates obtained in the real data analysis through this repetition process with 3 splits, we demonstrate how the number of repetitions affects the overall RD and corresponding SE estimates (Appendix Figure D.1). Here, we randomly sampled 1, 5, and 50 estimates without replacement from the 100 estimates and iterated the sampling process 10,000 times to generate the distribution of overall RDs and associated SEs, calculated using the method described earlier. The figure illustrates the benefits of more repetitions, as the corresponding distributions shrink with an increased number of repetitions. However, with more repetitions, the computational costs are significantly higher.

## 5 Discussion

### 5.1 Summary of Key Findings

#### 5.1.1 Scenarios under Consideration

In this work, we considered two scenarios in our simulation: one involving sample size and another involving two generalizations of DCF with higher folds. We used a data generating mechanism that utilizes complex patterns to generate data, and we also employed complex and flexible learners within the super learner to obtain this result. The DCF approach is supposed to be useful in this context.

#### 5.1.2 Performances in Terms of Bias, Variance, and Coverage

DCF with p=5 splits demonstrated satisfactory bias, variance, and coverage properties. Improvements in coverage and standard error calibration exceeded Monte Carlo uncertainty, while differences in bias were likely not statistically meaningful. However, patterns in performance measures such as relative error and coverage probabilities suggest that, when a larger cohort is available, the number of splits can potentially be reduced. Based on these results and after accounting for Monte Carlo variability, we found no reliable evidence that increasing the number of splits to p=10 or beyond provides statistically or practically better estimates.

When comparing Generalization 1 (equal splits) and Generalization 2 (full data), both performed best with the lowest relative error when considering p=5. This trend was consistent across coverage and bias‐eliminated coverage plots, where p=5 exhibited nearly nominal coverage. Generalization 2 has an obvious advantage in utilizing more data than Generalization 1 for propensity score and outcome model estimation. The amount of available data for Generalization 2 increases with the increase in splits. However, still, the performances of a higher number of splits p=9 was not better than p=5, particularly in terms of bias, average model SE, relative error, and coverage. This is likely due to the fact that with a higher number of splits, the data available for treatment effect estimation shrinks. However, using a greater number of splits is generally associated with a higher computing cost.

As previous research speculated, we show that subdividing the data (even for p = 9 or 10) too much may be detrimental for complex machine learning algorithms to provide reasonable results [9]. Still, we were able to demonstrate that an increment of split numbers (arguably up to a certain point, such as p=5) is helpful in obtaining desirable performance of the TMLE estimate. Increasing the number of splits is also associated with high computational costs, but may not necessarily provide better estimates and could be detrimental in terms of most statistical performance measures. On the contrary, while dealing with larger cohorts, we may even consider reducing the number of splits.

#### 5.1.3 Rationale of Exploring Generalization 1

One might intuitively anticipate that the performance of Generalization 1, in terms of bias, would worsen as the number of splits increases due to the reduced amount of data available for training in each split. This expectation stems from the direct relationship between the number of splits and the partition size of the dataset for cross‐validation‐type procedures. However, our analysis reveals a trade‐off: a smaller number of splits does not necessarily result in the least bias, as evidenced by the case for p=3. We also examined other performance measures such as standard errors and coverage probabilities, which yielded results that were not as intuitively predictable.

#### 5.1.4 Why Higher Number of Splits Can Still be an Issue for Generalization 2

In Generalization 1, it is easy to see that as the number of splits increases (e.g., in a 5‐split DCF), each model (both propensity and outcome models) is trained on a progressively smaller subset of the data (only 1 split), which limits the models' ability to capture complex relationships due to reduced exposure to diverse data patterns. While Generalization 2 refines this approach by fully utilizing all available non‐test data for model training (e.g., 2 splits for the propensity model and 2 splits for the outcome model in a 5‐split DCF setup), the predictions and the final treatment effect estimation for each repetition are still derived from a smaller portion of the data: the single dedicated split. This setup can potentially limit the amount of data available for estimating the treatment effect, which might affect the accuracy and reliability of those estimates.

#### 5.1.5 Our Recommendation Regarding the Generalizations

Our simulation results suggest that Generalization 2 yielded more stable and well‐calibrated variance estimates—even with fewer splits—likely due to its more efficient use of data for nuisance parameter estimation. Based on these findings, we recommend Generalization 2 as a practical and statistically robust default for analysts implementing DCF TMLE, particularly with a moderate number of splits (e.g., p=3 to 5). The approach is supported by the open‐source “Crossfit” “R”package, and its full‐data‐use strategy results in improved stability [33].

#### 5.1.6 Our Recommendation Regarding the Number of Repetitions

Our simulations indicate that increasing the number of repetitions from r=25 to r=100 provides minimal additional benefit: the resulting differences in bias, standard errors, and coverage were negligible (within three decimal places). Given the increased computational burden of higher r, we recommend r=25 as a reasonable and efficient choice in practice. Importantly, our results also suggest that the number of splits (p) has a more pronounced impact on performance than the number of repetitions.

While examining the densities of each performance measure (e.g., bias, SE) based on repetitions, r=100, we noted a few outliers. This observation highlights the problems associated with choosing a low number of repetitions (or r=1). One reason for the current underutilization of DCF in the literature is the computational burden associated with a high number of repetitions (r=100). However, our results demonstrate that the metrics from r=1 visibly differ from those obtained with moderate to high repetitions (r=25 to r=100). Therefore, we suggest practitioners use DCF with a moderate number of repetitions.

#### 5.1.7 Considerations for Smaller Datasets

Our simulations focused on sample sizes of 3000 or larger. In real‐world applications involving smaller datasets, the trade‐off between bias and variance becomes more pronounced. In such settings, increasing the number of splits beyond three may be detrimental, as each fold contains less data for estimating the target parameter, potentially inflating variance. We therefore recommend that analysts carefully consider both sample size and the complexity of machine learning algorithms when selecting the number of splits in the DCF framework.

#### 5.1.8 Real‐World Analysis

In our real‐world analysis of a sample size of n=2418 from the NHANES 2017–18 cycle, we found that the estimated risk differences (RD) for DCF TMLEs remained stable across different numbers of splits. However, the standard errors increased as the number of splits grew in Generalization 1. Additionally, the computation time for Generalization 2 increased exponentially with an increasing number of splits. Individual results from each repetition in our real data analysis were also analyzed, which predictably shows some variability in the results with the choice of smaller repetitions.

### 5.2 Limitations

Indeed, work related to doubly robust approach involving machine learning methods represents a burgeoning area of research, yet our current work is not without limitations. This simulation study was conducted in a low‐dimensional setting, considering only a few covariates, which may not fully capture the complexities of many real‐world scenarios [30]. Our real‐world analysis included large number of covariates and proxies, and the standard error patterns from this analysis look similar to those observed in the simulations. There are other ongoing research initiatives that explore how estimates behave in simulations with a higher number of covariates [42]. In our study, we utilized the RD as a measure of effect, which resulted in bias estimates appearing smaller. Future research should consider a broader range of effect estimates. Moreover, in our work, we applied Generalization 2 only in scenarios with an odd number of splits (e.g., 9 splits), allocating one split for treatment effect estimation while distributing the remaining folds equally for propensity score and outcome model estimation. However, adapting this method to situations with an even number of splits should be straightforward. In scenarios with an even number of splits, one split can be reserved for treatment effect estimation, while the data from the remaining odd number of splits can be divided equally for propensity score and outcome model estimation. Please see Appendix E for additional limitations and future research directions.

### 5.3 Implication

The findings of our study highlight the importance of carefully selecting the number of splits and repetitions when employing DCF TMLE methods. This choice is pivotal in harnessing the full potential of complex machine learning algorithms while ensuring robust and valid statistical inference, as well as computational efficiency. These insights can provide valuable guidance for researchers seeking to leverage DCF TMLE in their causal investigations.

## Author Contributions

**Mohammad Ehsanul Karim:** conceptualization, supervision, updating analysis, preparing manuscript figures, writing – original draft, review and editing. **Momenul Haque Mondol:** software coding, executing analysis on the server, gathering results, and review manuscript.

## Ethics Statement

Ethics for this study was covered by item 7.10.3 in University of British Columbia's Policy #89: Research and Other Studies Involving Human Subjects 19 and Article 2.2 in of the Tri‐Council Policy Statement: Ethical Conduct for Research Involving Humans (TCPS2).

## Consent

The National Health and Nutrition Examination Survey (NHANES), conducted by the U.S. Centers for Disease Control and Prevention (CDC), involves collecting data through direct physical examinations, laboratory testing, and interviews. The CDC already obtains consent from participants when collecting this data. When researchers use NHANES data for their studies, they are typically using de‐identified, publicly available data. This means that the information cannot be linked back to individual participants, and therefore, additional consent from participants is not required for researchers to use this data. Given the secondary (from SUPPORT study) and anonymized nature of the data, individual consent for publication is not applicable. All results are presented in aggregate form, and no individual data are disclosed in this publication.

## Conflicts of Interest

Over the past 3 years, Mohammad Ehsanul Karim has received consulting fees from Biogen Inc. for consulting unrelated to this current work. Momenul Haque Mondol declares no conflict.

## Supporting information


**Data S1:** Supplementary Information.

## Data Availability

NHANES data is publicly accessible and can be retrieved from the NHANES website. The datasets generated and/or analyzed during the current study can be requested from the authors. The software codes can be accessed in the second author's GitHub repository [33]. Any use of the provided code should be cited appropriately in subsequent publications or presentations.
