# Supplementary material for: Finding the Optimal Number of Splits and Repetitions in Double Cross‐Fitting Targeted Maximum Likelihood Estimators
Source: Pharm Stat. 2025 Sep 11;24(5):e70022. doi: 10.1002/pst.70022 (PMC12425639; doi:10.1002/pst.70022)
Supplement: Supplementary file 1 — Data S1: Supplementary Information. [file PST-24-0-s001.pdf]

Supplementary Content "Finding the Optimal Number of Splits and Repetitions in Double Cross-Fitting Targeted Maximum Likelihood Estimators"

A Explanations of Structural Assumptions

A.1 Donsker Class

The Donsker class condition originates from empirical process theory and refers to a requirement on the complexity of the function class used for estimation (van der Vaart and Wellner, 1996). In intuitive terms, it asks whether the learning algorithm or estimator behaves in a regular and predictable way as the sample size grows. If the function class is too flexible (e.g., deep trees, neural networks, or certain ensemble learners), it may violate the Donsker condition, making standard inference (e.g., confidence intervals) invalid unless special techniques—such as sample splitting or cross-fitting—are used to restore validity (Chernozhukov et al., 2018; Zheng and van der Laan, 2010).

Simpler estimators (e.g., linear models, regression splines) often belong to Donsker classes by default, which is why they are sometimes preferred in finite samples to avoid the need for cross-fitting (Balzer and Westling, 2021).

A.2 Hölder Smoothness

Hölder smoothness is a mathematical condition that quantifies how smooth a function is—not just whether it is continuous, but how gradually it can change. Specifically, a function with Hölder smoothness of order $\alpha \in (0, 1]$ cannot change too abruptly; its rate of change is bounded, limiting how steeply it can curve at any point.

In statistical applications, assuming that nuisance functions—such as the treatment assignment model or outcome regression—satisfy Hölder smoothness means they vary in a controlled, predictable way and can be well-approximated by simpler functions. This structure enables the use of more advanced estimation strategies—such as higher-order influence function estimators or undersmoothing techniques—which help reduce bias, improve precision, and achieve faster convergence rates for target parameter estimates (Newey et al., 1998; Robins et al., 2008; Giné and Nickl, 2008; Balakrishnan et al., 2023).

These structural assumptions are often discussed when trying to achieve statistically optimal results (e.g., minimax-optimal inference) in settings where we want to avoid strong parametric assumptions (e.g., linearity) and use more flexible nonparametric or semiparametric models.

B Data Generation Mechanism

Following is the data generating mechanism as described in Zivich and Breskin (2021):

Covariates

Covariates $L = (G, N, D, F, R)$:

1. G: age,
2. N: natural-log transformed low-density lipoprotein,

3. D: diabetes,
4. F: frailty, and
5. R: risk score.

Also define $\text{expit}(.) = \frac{\exp(.)}{1+\exp(.)}$.

(1) Age

First define x:

$$x = \frac{55 \times \text{Uniform}(0,1) + 80}{2}$$

Then generate age of each person (i) as follows: the distribution of age is generated using a variable x, with specific conditions determining age (G).

$$G_i = \begin{cases} 75 - \sqrt{30(x_i - 60)} & \text{if } x_i > 60 \\ x_i & \text{if } x_i \leq 60 \end{cases}$$

(2) Low-density lipoprotein (natural-log transformed)

The value of low-density lipoprotein (N) is calculated based on age.

$$N_i = 0.005G_i + \text{Normal}(\log(100), 0.18)$$

(3) Diabetes

The status of diabetes (D) is determined using a logistic function involving age and low-density lipoprotein.

$$D_i = \text{Bernoulli}\left(\text{expit}\left(-4.23 + 0.03N_i - 0.02G_i + 0.0009G_i^2\right)\right)$$

(4) Frailty

Frailty (F) is generated using a logistic function involving age.

$$F_i = \text{expit}\left(-5.5 + 0.05(G_i - 20) + 0.001G_i^2 + \text{Normal}(0,1)\right)$$

(5) Risk score

The risk score (R) is calculated through a logistic function involving diabetes status, age, low-density lipoprotein, and frailty.

$$R_i = \text{expit}\Bigg(4.299 + 3.501 D_i - 2.07\log(G_i) +$$

$$0.051\log(G_i)^2 + 4.090 N_i - 1.04\log(G_i) N_i + 0.01 F_i\Bigg)$$

Treatment

Treatment assignment (A) is determined using a logistic function involving diabetes status, age, low-density lipoprotein and risk score.

$$\begin{aligned}\Pr(A|L) = \text{Bernoulli}\,(&\text{expit}\,(-3.471 + 1.390 D_i + 0.112 N_i \\ &+0.973 I(N_i > \ln(60)) - 0.046(G_i - 30) + 0.003(G_i - 30)^2 \\ &+0.273 I(0.05 \leq R_i < 0.075) + 1.592 I(0.075 \leq R_i < 0.2) + \\ &2.641 I(R_i \geq 0.2)))\end{aligned}$$

Potential Outcome

The potential outcome (Y^a) is generated using a logistic function with a formula that involves treatment, diabetes status, age, low-density lipoprotein and risk score.

$$\begin{aligned}\Pr(Y^a|a, L) = \text{Bernoulli}\,(&\text{expit}\,(-6.25 - 0.75a + \\ &0.35a(5 - N_i) I(N_i < \ln(130)) + \\ &0.45(G_i - 39)^{0.5} + \\ &1.75 D_i + 0.29\exp(R_i + 1) + \\ &0.14 I(N_i > \ln(120)) N_i^2))\end{aligned}$$

Here, by exchangeability, P(Y^a=1|a, L) = P(Y^a=1|L).

Outcome

The observed outcome (Y) is generated.

$$Y_i = A_i Y_i^1 + (1 - A_i) Y_i^0$$

Treatment effect of interest

The treatment effect of interest (ψ) is the expected difference between potential outcomes under different treatments.

$$\psi = E[Y^1 - Y^0]$$

To calculate the value for ψ (true value, in order to calculate bias), the potential outcomes of $10,000,000$ individuals were generated.

C Performance Measures in Simulations

Further Details

Detailed performance measures of each simulation are presented in Appendix C (Appendix Figures C.1-C.7 for different sample sizes, Appendix Figures C.8-C.14 for different generalizations and Appendix Figures C.15-C.21 for different number of repetitions). Interactive plots, including individual simulation results with additional number of splits (p) considered in some cases, can be viewed as a Shiny App by executing the following command in RStudio: `shiny::runGitHub("dcf", "ehsanx")`. Please note that executing this command requires the installation of the `shiny` package and an internet connection to download the shiny app from GitHub. The results and figures presented in the app can also be fully accessed and reproduced using the 'R' scripts and data available at: https://github.com/ehsanx/dcf. The software package implementing the double cross-fitting procedures is available at: https://github.com/momenulhaque/Crossfit (Mondol and Karim, 2023, 2024). To aid interpretation, we reported MC SEs alongside each performance metric in Appendix Tables C.1-C.4, allowing readers to assess whether the observed differences between estimators are meaningful or within the range of simulation variability.

Different Sample Sizes

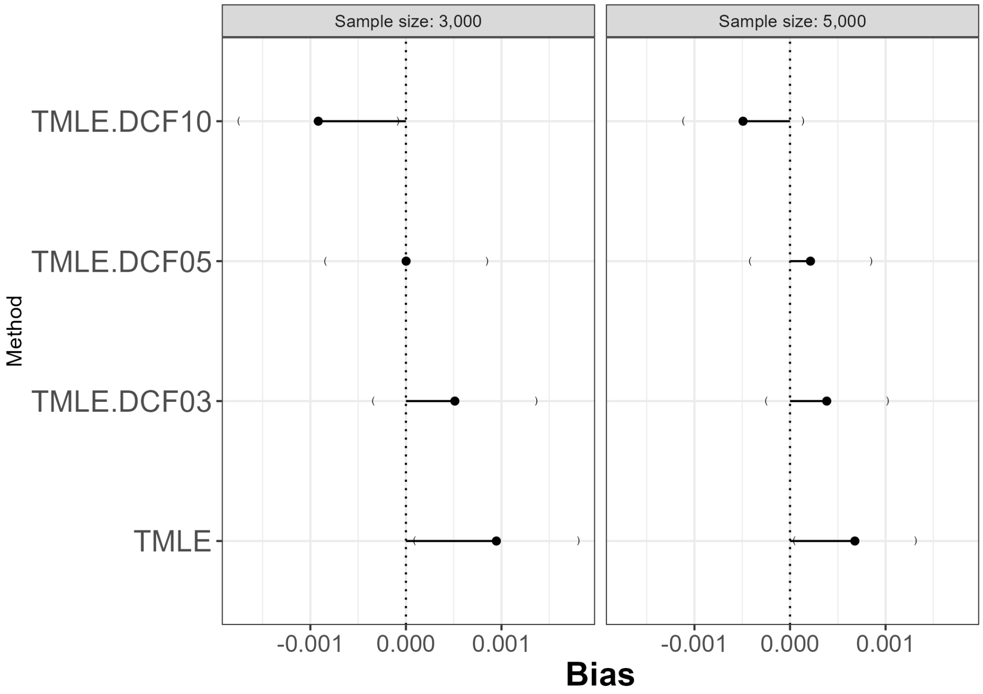

Appendix Figure C.1: Simulation results comparing the bias under two different sample sizes under Generalization 1.

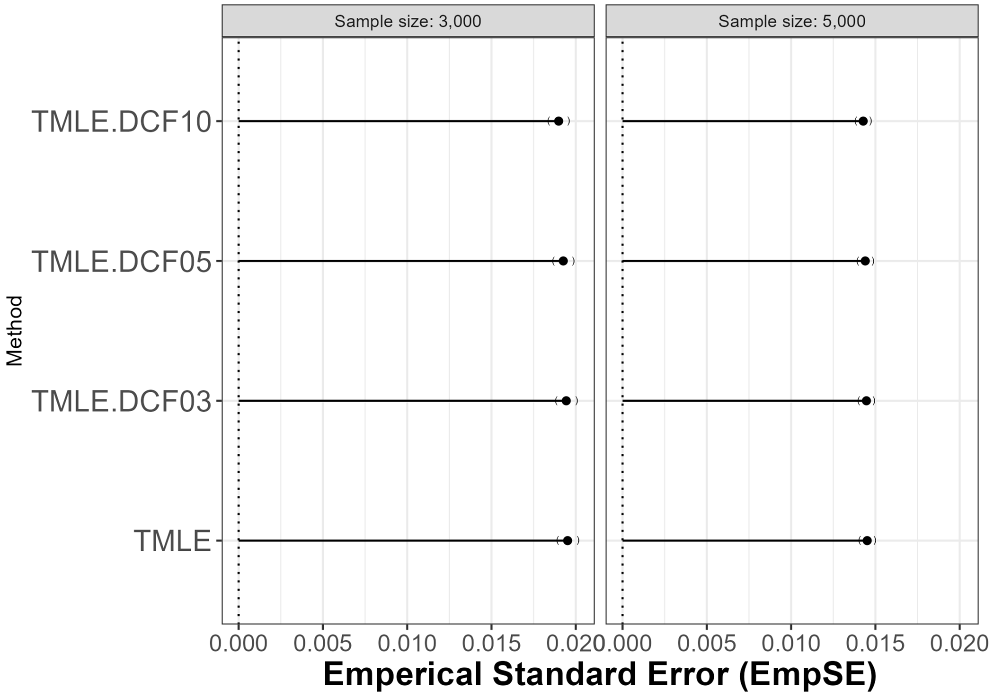

Appendix Figure C.2: Simulation results comparing the empirical standard error under two different sample sizes under Generalization 1.

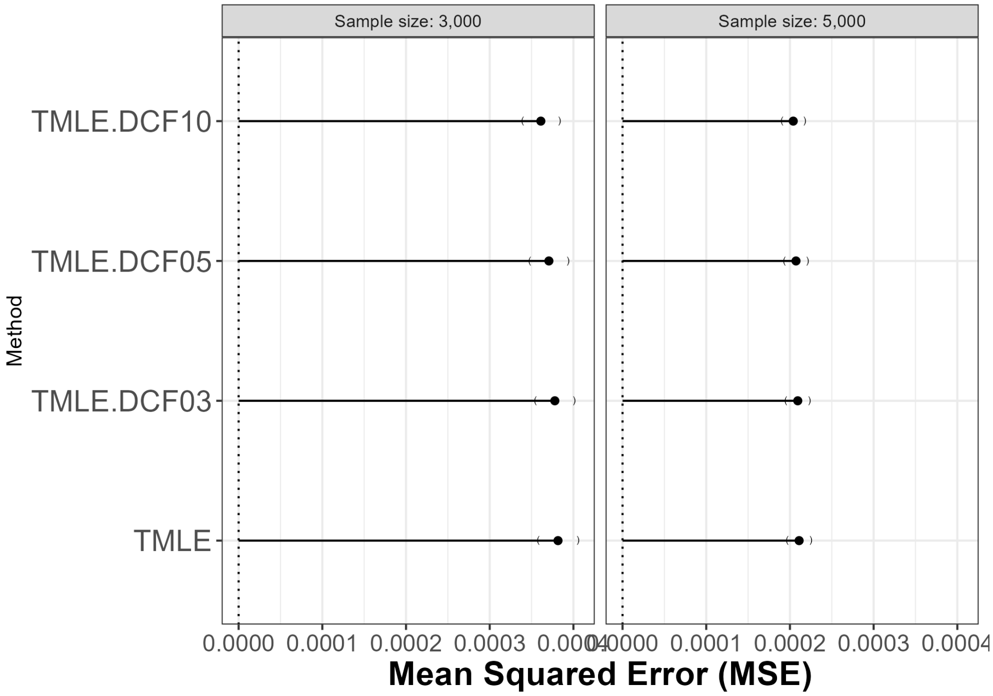

Appendix Figure C.3: Simulation results comparing the mean squared error under two different sample sizes under Generalization 1.

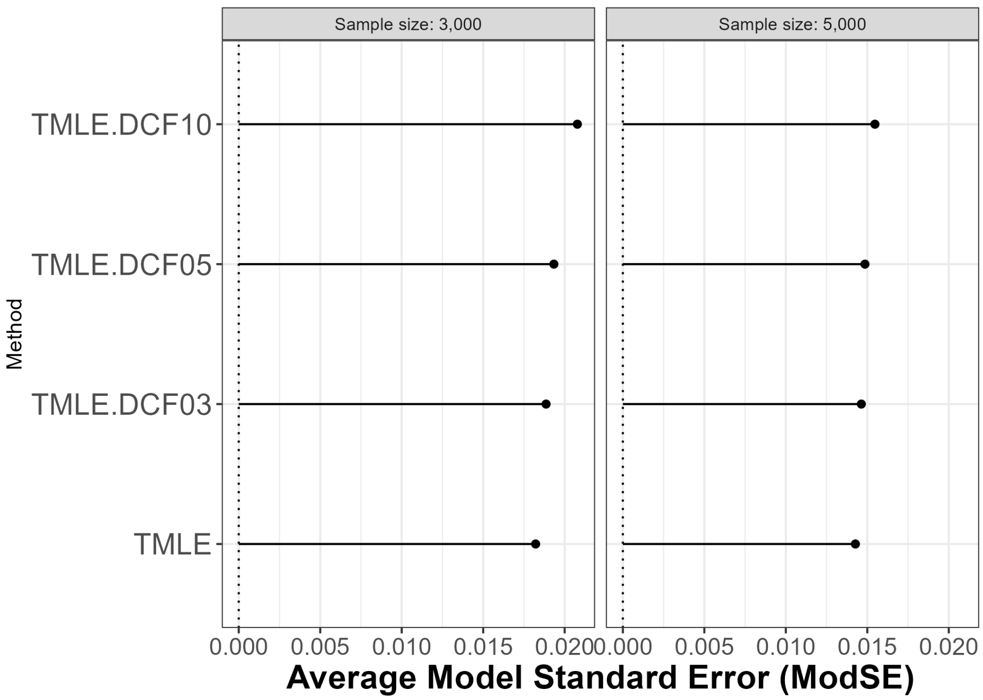

Appendix Figure C.4: Simulation results comparing the model standard error under two different sample sizes under Generalization 1.

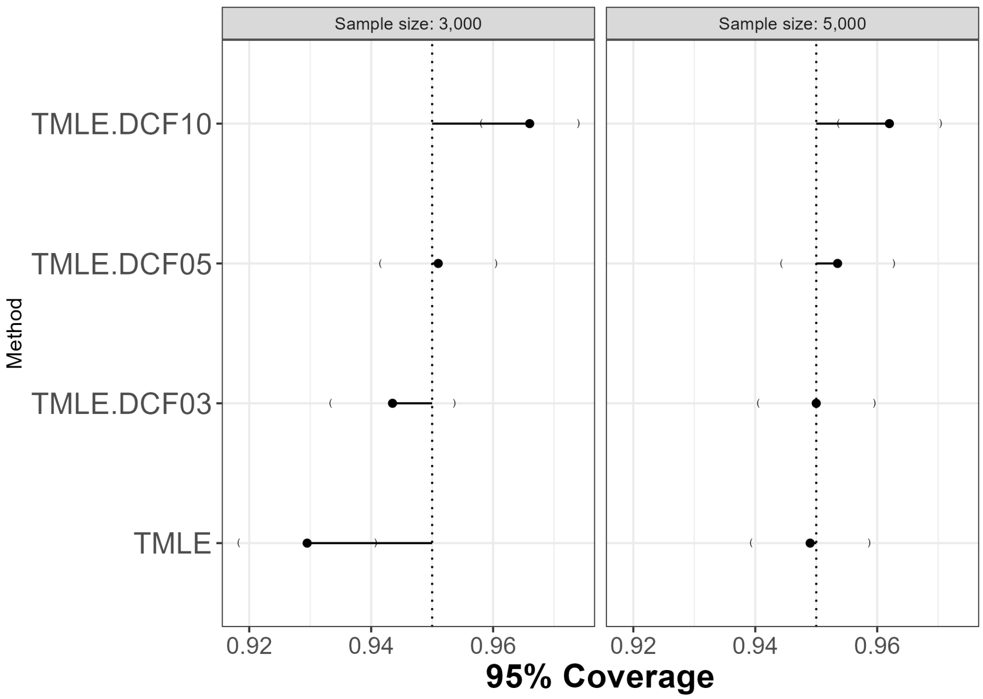

Appendix Figure C.5: Simulation results comparing the coverage probability of 95% confidence intervals under two different sample sizes under Generalization 1.

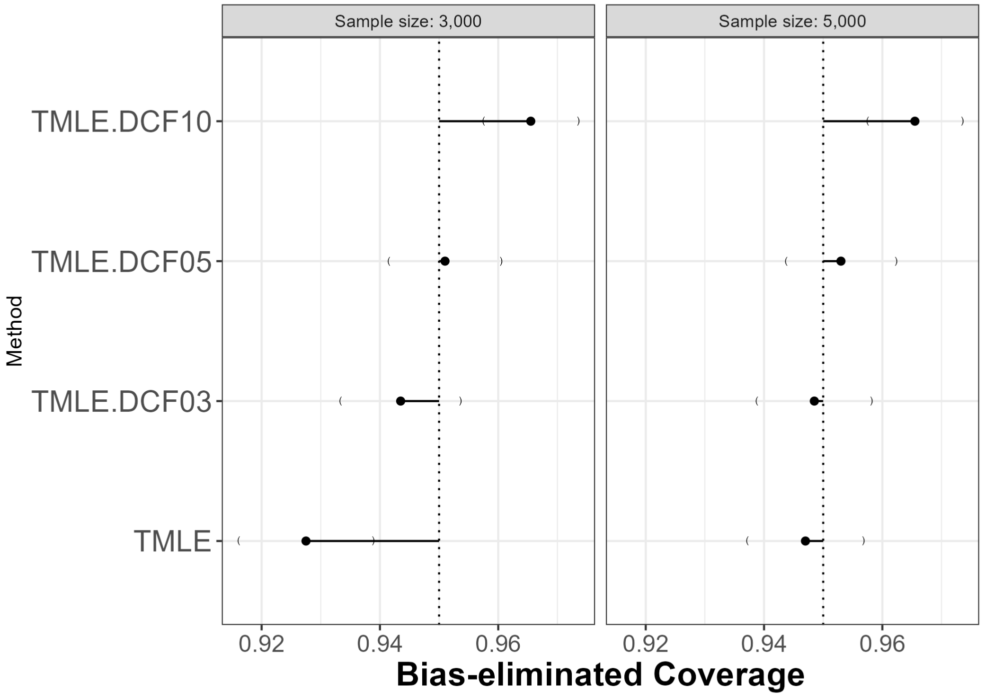

Appendix Figure C.6: Simulation results comparing the bias-eliminated coverage under two different sample sizes under Generalization 1.

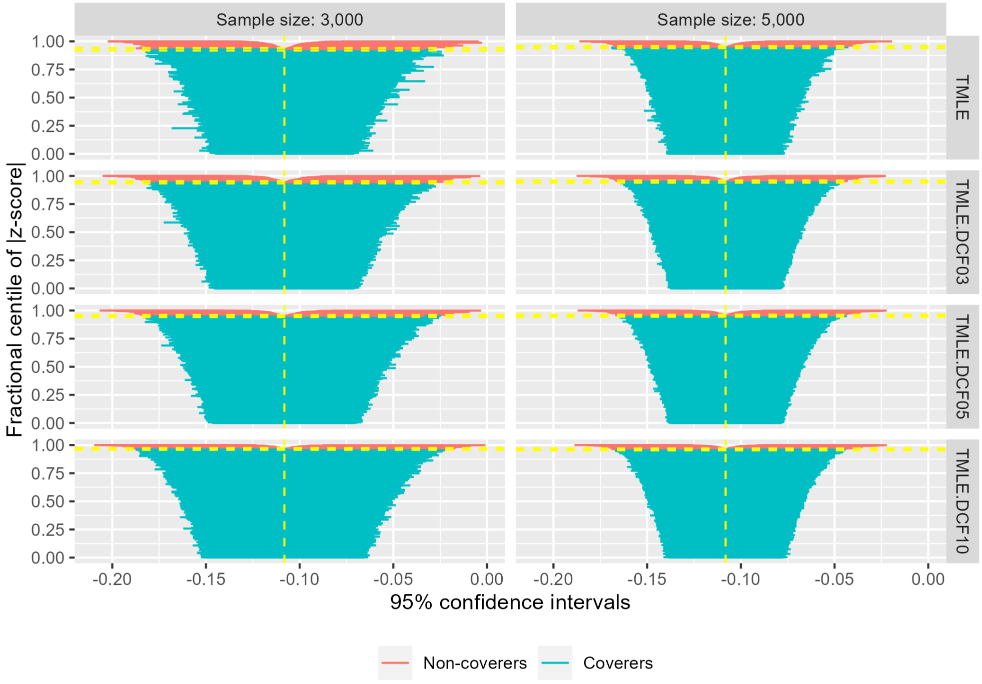

Appendix Figure C.7: Simulation results comparing the Zip plot under two different sample sizes under Generalization 1. Zip plot helps visualize the coverage properties of confidence intervals across different methods. The x-axis represents the range of 95 percent confidence intervals for the estimated treatment effects, while the y-axis represents the fractional centiles of the z-score, providing a standardized comparison across methods. The blue regions indicate cases where the confidence interval contains the true treatment effect (coverage), whereas the red regions represent cases where the confidence interval fails to cover the true value. The yellow dashed line represents the expected nominal 95 percent coverage level. A well-calibrated method should have confidence intervals symmetrically distributed around this line, with minimal red areas at the edges. This plot allows us to assess the reliability of different methods in achieving nominal coverage and compare the impact of varying the number of splits in DCF TMLE.

Different Generalizations of DCF

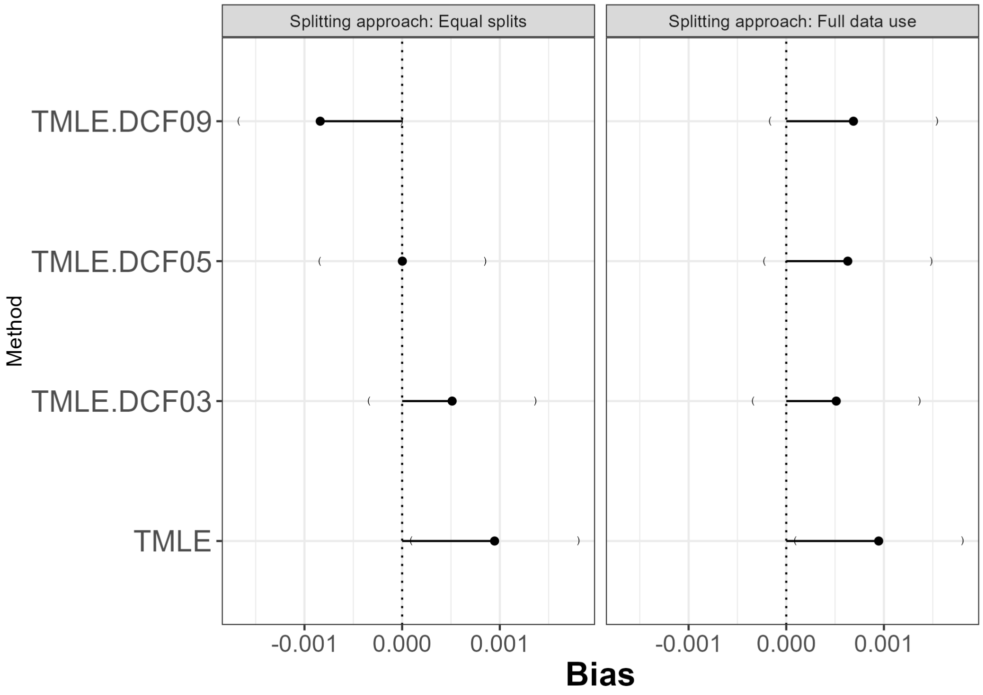

Appendix Figure C.8: Simulation results comparing the bias under two different generalizations for sample size 3,000.

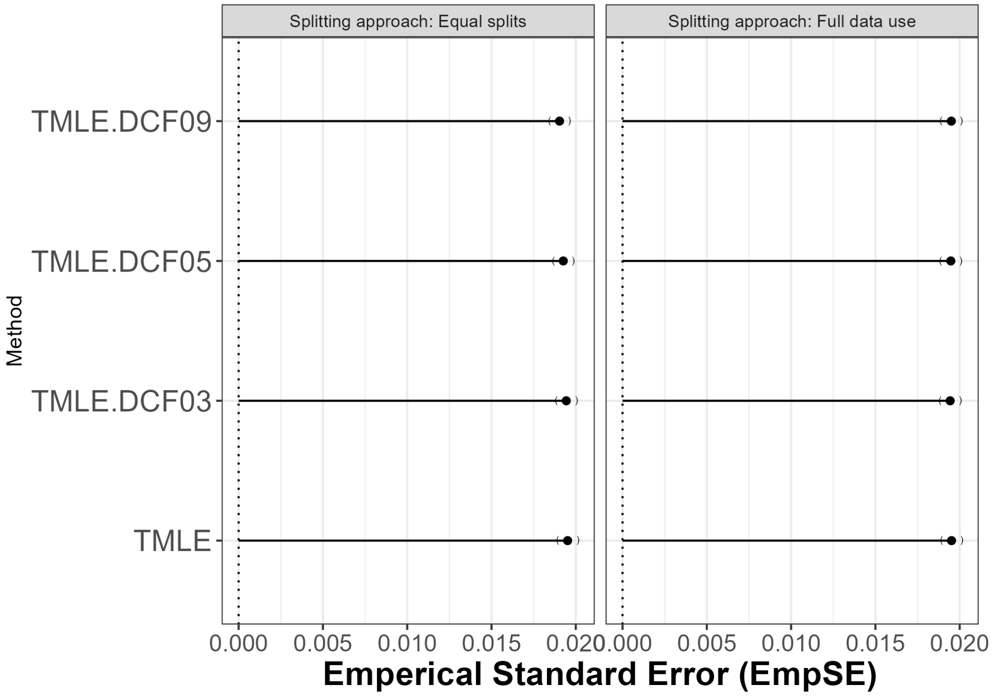

Appendix Figure C.9: Simulation results comparing the empirical standard error under two different sample sizes for sample size 3,000.

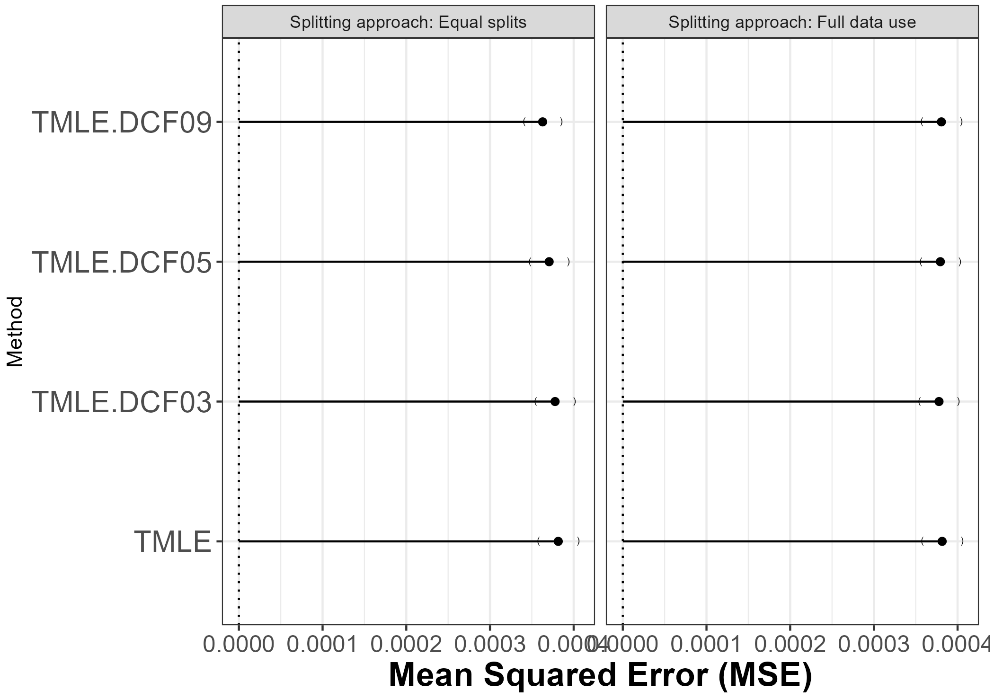

Appendix Figure C.10: Simulation results comparing the mean squared error under two different generalizations for sample size 3,000.

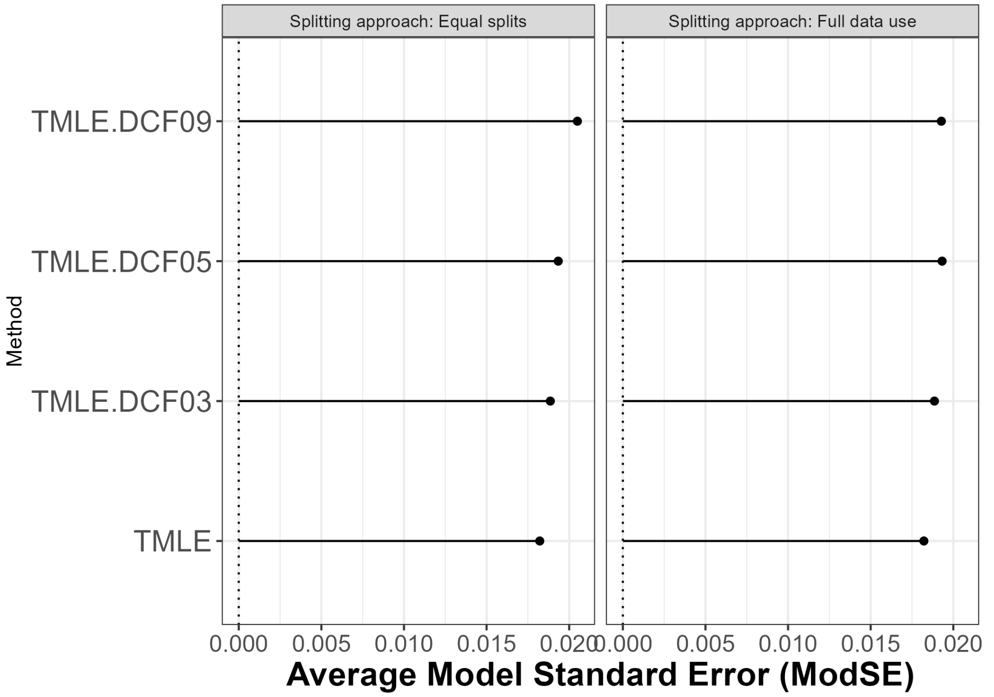

Appendix Figure C.11: Simulation results comparing the model standard error under two different generalizations for sample size 3,000.

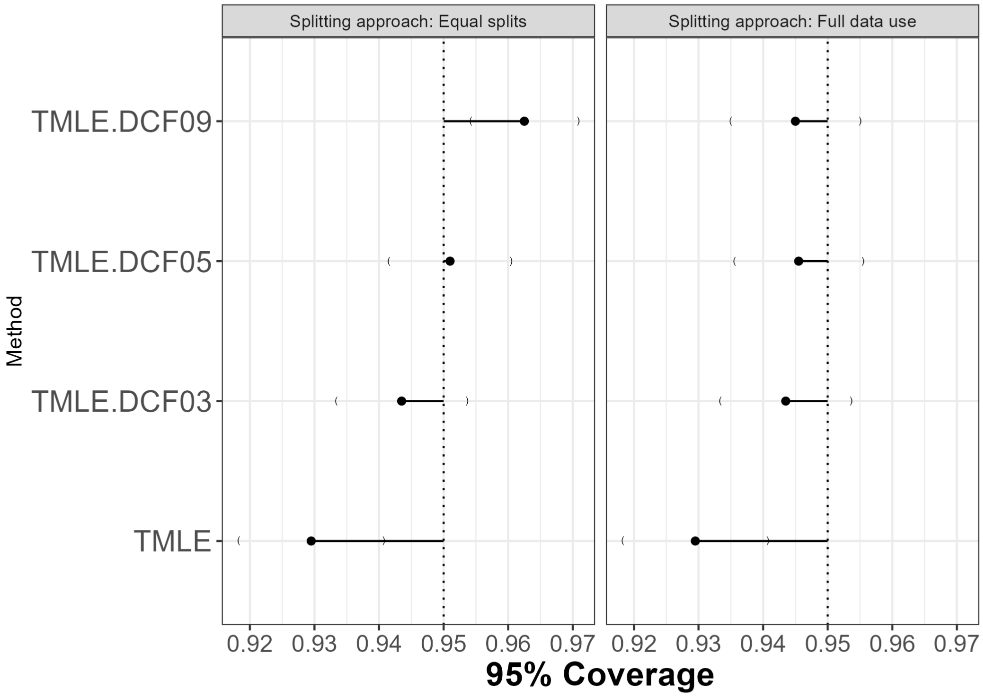

Appendix Figure C.12: Simulation results comparing the coverage probability of 95% confidence intervals under two different generalizations for sample size 3,000.

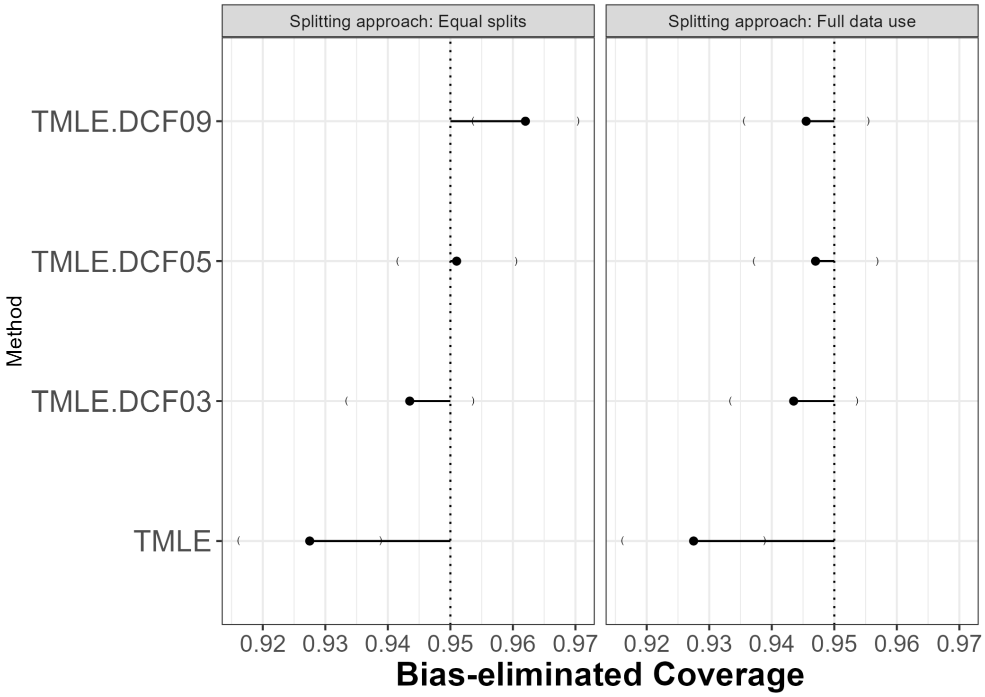

Appendix Figure C.13: Simulation results comparing the bias-eliminated coverage under two different generalizations for sample size 3,000.

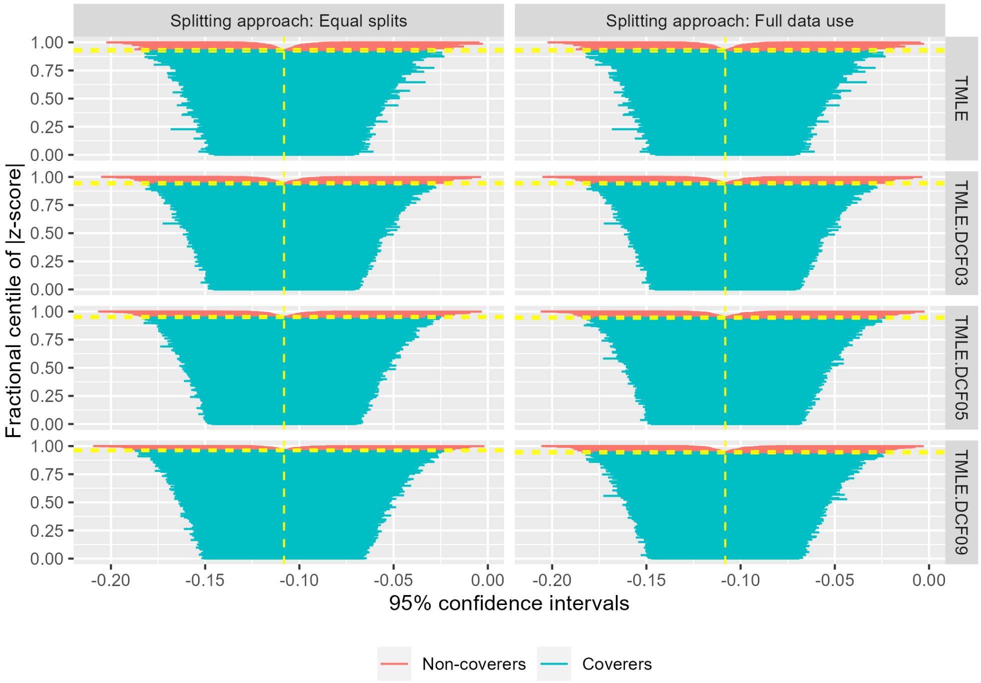

Appendix Figure C.14: Simulation results comparing the Zip plot under two different generalizations for sample size 3,000.

Different Number of Repetitions

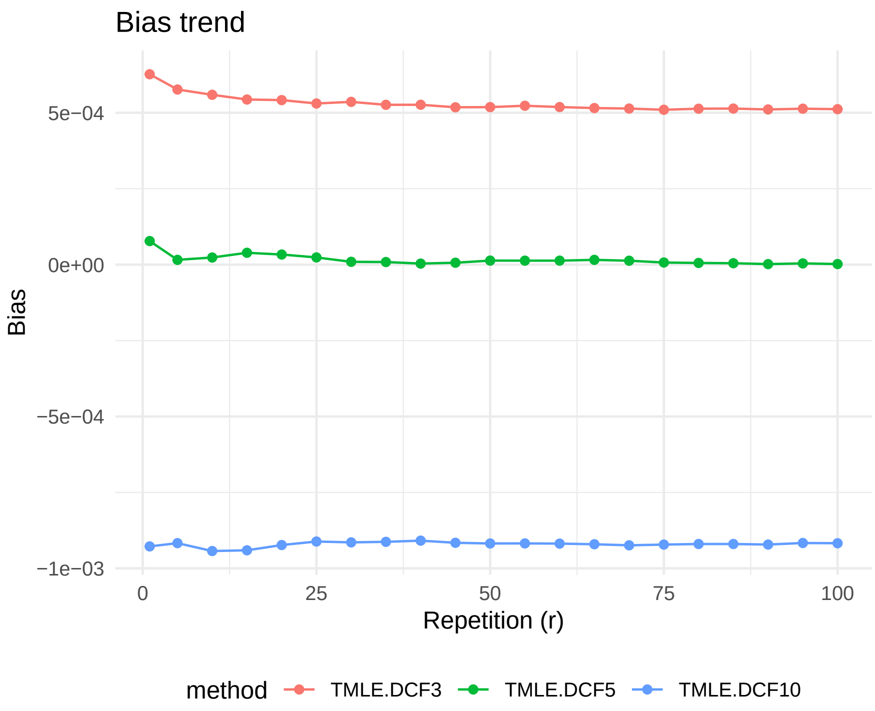

Appendix Figure C.15: Simulation results comparing the bias for base simulation settings (Generalization 1 and Sample size 3,000) with different number of repetitions.

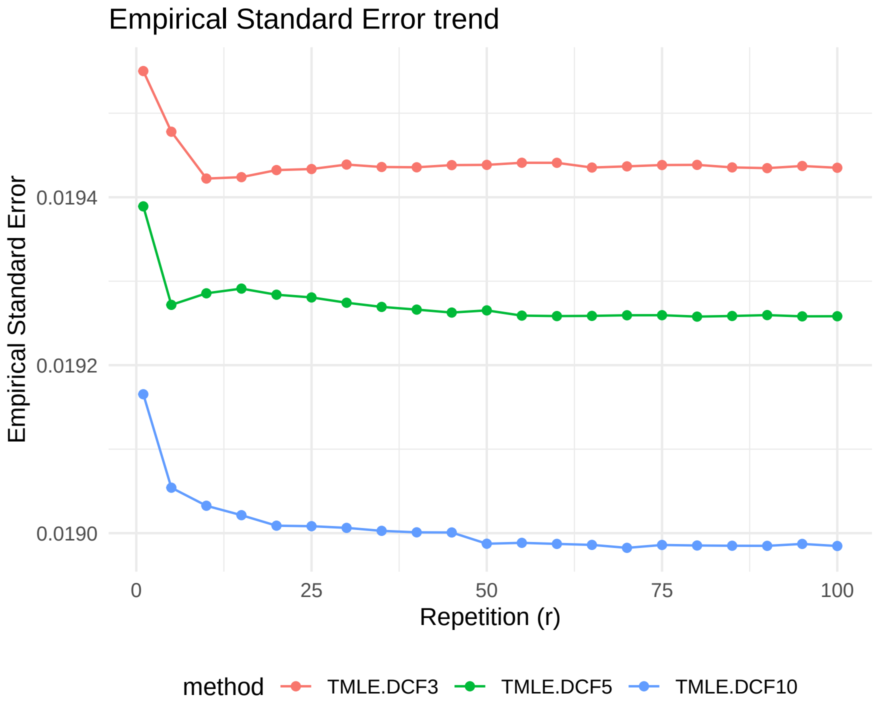

Appendix Figure C.16: Simulation results comparing the empirical standard error for base simulation settings (Generalization 1 and Sample size 3,000) with different number of repetitions.

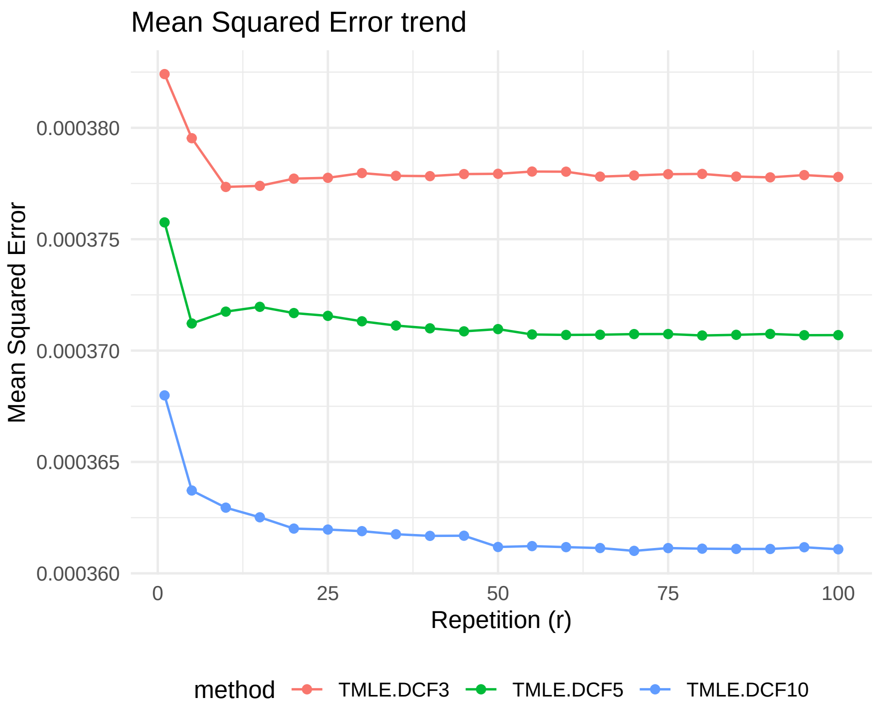

Appendix Figure C.17: Simulation results comparing the mean squared error for base simulation settings (Generalization 1 and Sample size 3,000) with different number of repetitions.

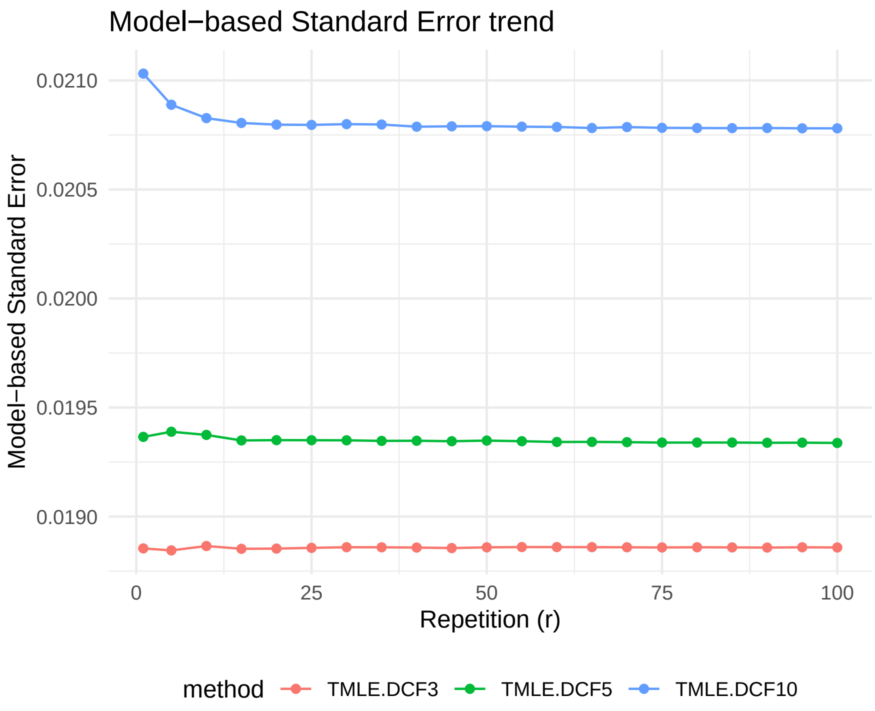

Appendix Figure C.18: Simulation results comparing the model standard error for base simulation settings (Generalization 1 and Sample size 3,000) with different number of repetitions.

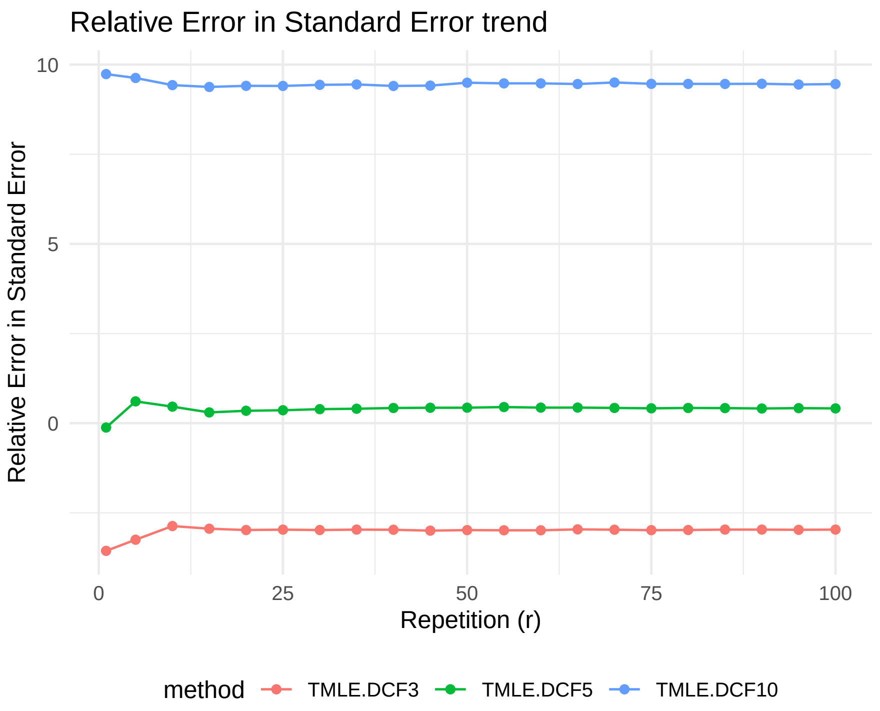

Appendix Figure C.19: Simulation results comparing the relative errors in model standard errors for base simulation settings (Generalization 1 and Sample size 3,000) with different number of repetitions.

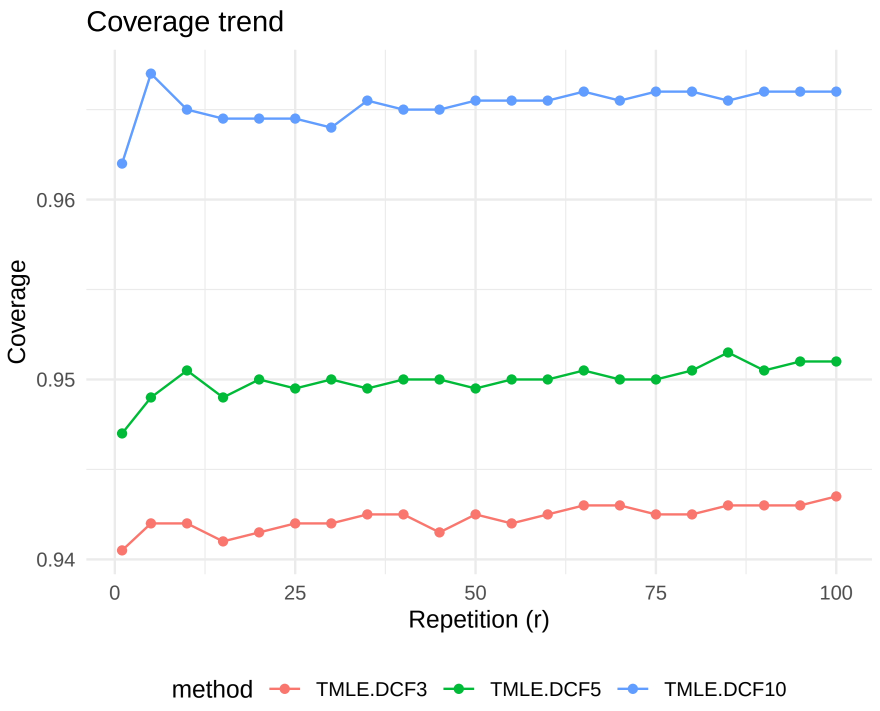

Appendix Figure C.20: Simulation results comparing the coverage probability of 95% confidence intervals for base simulation settings (Generalization 1 and Sample size 3,000) with different number of repetitions.

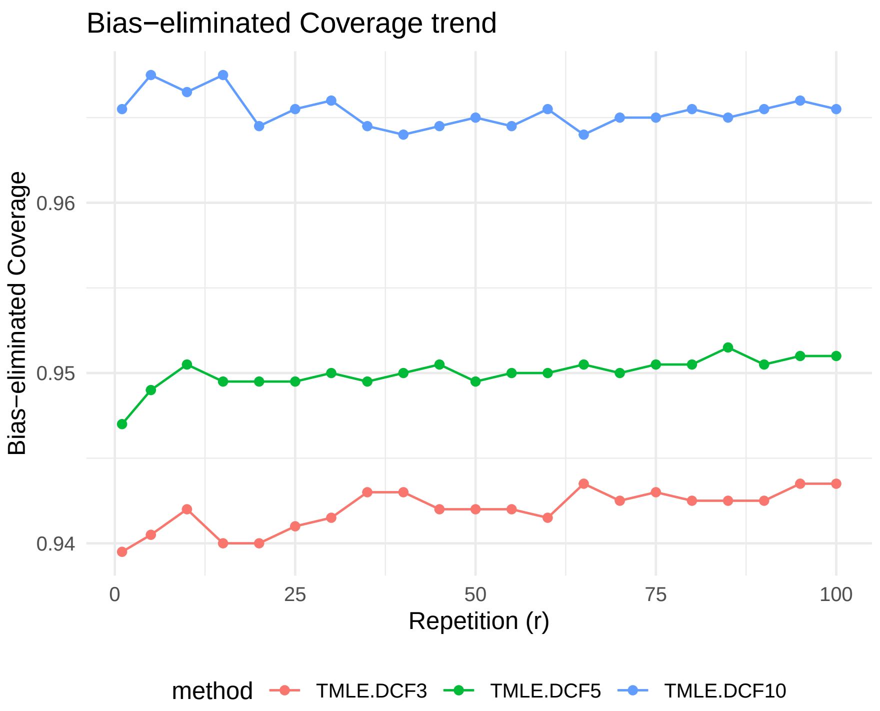

Appendix Figure C.21: Simulation results comparing the bias-eliminated coverage for base simulation settings (Generalization 1 and Sample size 3,000) with different number of repetitions.

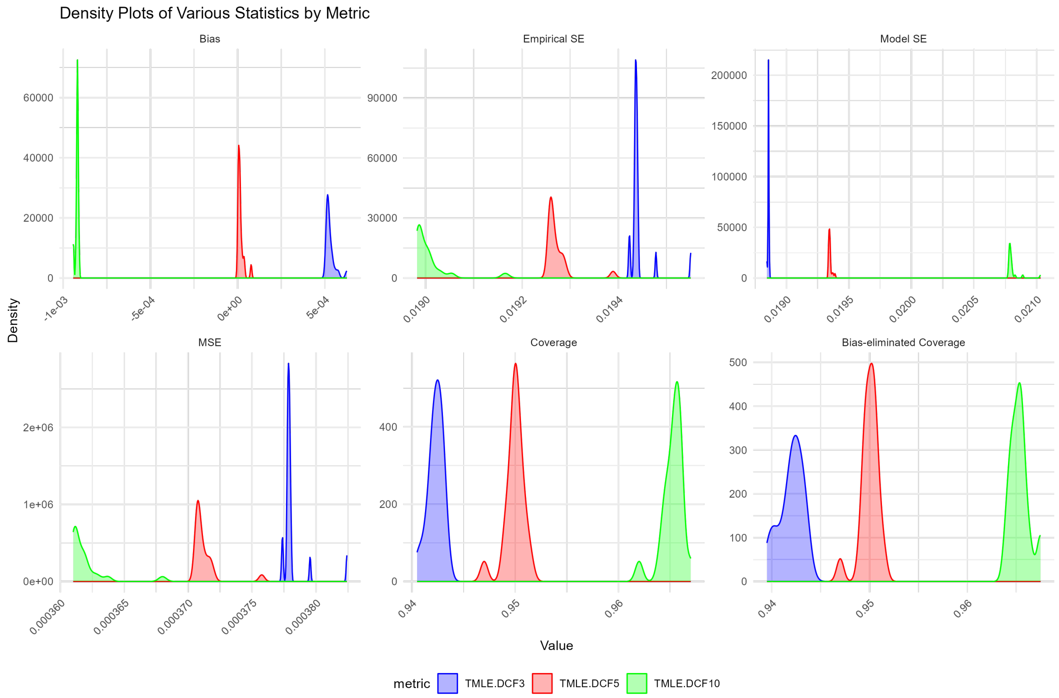

Appendix Figure C.22: The density plots of the distribution of various statistical metrics—Bias, Empirical Standard Error (Empirical SE), Model Standard Error (Model SE), Mean Squared Error (MSE), Coverage, and Bias-eliminated Coverage—across different methods of analysis (TMLE.DCF3, TMLE.DCF5, TMLE.DCF10) for Generalization 1 and Sample size 3,000.

Simulation Results in Numerical Form

- TMLE: Standard Targeted Maximum Likelihood Estimator without double cross-fitting (DCF).
- TMLE.DCF03: TMLE with Double Cross-Fitting using 3 splits ($p = 3$).
- TMLE.DCF05: TMLE with Double Cross-Fitting using 5 splits ($p = 5$).
- TMLE.DCF10: TMLE with Double Cross-Fitting using 10 splits ($p = 10$).

C.1 Sample Size 3,000 for Generalization 1

Appendix Table C.1: Simulation performance metrics for TMLE and DCF TMLE methods under Generalization 1 with sample size $n = 3{,}000$. Values in parentheses represent Monte Carlo standard errors. Metrics include bias, precision, standard errors, confidence interval coverage, and power.

Metric	TMLE	TMLE.DCF03	TMLE.DCF05	TMLE.DCF10
Bias in point estimate	0.0009 (0.0004)	0.0005 (0.0004)	0.0000 (0.0004)	-0.0009 (0.0004)
Relative bias in point estimate	-0.0088 (0.0040)	-0.0047 (0.0040)	-0.0000 (0.0040)	0.0085 (0.0039)
Empirical standard error	0.0195 (0.0003)	0.0194 (0.0003)	0.0193 (0.0003)	0.0190 (0.0003)
% Gain in precision vs TMLE	0.0000 (0.0000)	0.8354 (4.5085)	2.6952 (4.5905)	5.6762 (4.7135)
Mean squared error	0.0004 (0.0000)	0.0004 (0.0000)	0.0004 (0.0000)	0.0004 (0.0000)
Model-based standard error	0.0182 (0.0000)	0.0189 (0.0000)	0.0193 (0.0000)	0.0208 (0.0000)
Relative % error in SE	-6.6489 (1.4844)	-2.9672 (1.5384)	0.4122 (1.5918)	9.4588 (1.7352)
Coverage of nominal 95% CI	0.9295 (0.0057)	0.9435 (0.0052)	0.9510 (0.0048)	0.9660 (0.0041)
Bias-eliminated 95% CI coverage	0.9275 (0.0058)	0.9435 (0.0052)	0.9510 (0.0048)	0.9655 (0.0041)
Power of 5% level test	1.0000 (0.0000)	1.0000 (0.0000)	1.0000 (0.0000)	1.0000 (0.0000)

C.2 Sample Size 5,000 for Generalization 1

Appendix Table C.2: Simulation performance metrics for TMLE and DCF TMLE methods under Generalization 1 with sample size $n = 5{,}000$. Monte Carlo standard errors are reported in parentheses to assess estimation uncertainty.

Metric	TMLE	TMLE.DCF03	TMLE.DCF05	TMLE.DCF10
Bias in point estimate	0.0007 (0.0003)	0.0004 (0.0003)	0.0002 (0.0003)	-0.0005 (0.0003)
Relative bias in point estimate	-0.0063 (0.0030)	-0.0036 (0.0030)	-0.0020 (0.0030)	0.0045 (0.0030)
Empirical standard error	0.0145 (0.0002)	0.0145 (0.0002)	0.0144 (0.0002)	0.0143 (0.0002)
% Gain in precision vs TMLE	0.0000 (0.0000)	0.6308 (4.5013)	1.6027 (4.5447)	3.3405 (4.6225)
Mean squared error	0.0002 (0.0000)	0.0002 (0.0000)	0.0002 (0.0000)	0.0002 (0.0000)
Model-based standard error	0.0143 (0.0000)	0.0146 (0.0000)	0.0149 (0.0000)	0.0155 (0.0000)
Relative % error in SE	-1.6773 (1.5604)	1.1497 (1.6023)	3.1830 (1.6344)	8.3453 (1.7161)
Coverage of nominal 95% CI	0.9490 (0.0049)	0.9500 (0.0049)	0.9535 (0.0047)	0.9620 (0.0043)
Bias-eliminated 95% CI coverage	0.9470 (0.0050)	0.9485 (0.0049)	0.9530 (0.0047)	0.9655 (0.0041)
Power of 5% level test	1.0000 (0.0000)	1.0000 (0.0000)	1.0000 (0.0000)	1.0000 (0.0000)

C.3 Sample Size 3,000 for Generalization 2

Appendix Table C.3: Simulation performance metrics for TMLE and DCF TMLE methods under Generalization 2 with sample size $n = 3{,}000$. Results are shown with Monte Carlo standard errors in parentheses, reflecting the impact of using full data for model estimation in each repetition.

**Metric**	**TMLE**	**TMLE.DCF03**	**TMLE.DCF05**	**TMLE.DCF09**
Bias in point estimate	0.0009 (0.0004)	0.0005 (0.0004)	0.0006 (0.0004)	0.0007 (0.0004)
Relative bias in point estimate	-0.0088 (0.0040)	-0.0047 (0.0040)	-0.0058 (0.0040)	-0.0064 (0.0040)
Empirical standard error	0.0195 (0.0003)	0.0194 (0.0003)	0.0195 (0.0003)	0.0195 (0.0003)
% Gain in precision vs TMLE	0.0000 (0.0000)	0.8354 (4.5085)	0.4385 (4.4898)	0.1281 (4.4760)
Mean squared error	0.0004 (0.0000)	0.0004 (0.0000)	0.0004 (0.0000)	0.0004 (0.0000)
Model-based standard error	0.0182 (0.0000)	0.0189 (0.0000)	0.0193 (0.0000)	0.0193 (0.0000)
Relative % error in SE	-6.6489 (1.4844)	-2.9672 (1.5384)	-0.7740 (1.5736)	-1.1594 (1.5676)
Coverage of nominal 95% CI	0.9295 (0.0057)	0.9435 (0.0052)	0.9455 (0.0051)	0.9450 (0.0051)
Bias-eliminated 95% CI coverage	0.9275 (0.0058)	0.9435 (0.0052)	0.9470 (0.0050)	0.9455 (0.0051)
Power of 5% level test	1.0000 (0.0000)	1.0000 (0.0000)	1.0000 (0.0000)	1.0000 (0.0000)

C.4 Summarizing the information from 3 Simulation Results

Appendix Table C.4: Interpretation of Simulation Results Based on Monte Carlo Standard Errors (MC SEs)

**Metric**	**Generalization 1 Sample Size 3,000**	**Generalization 1 Sample Size 5,000**	**Generalization 2 Sample Size 3,000**
Bias	Differences range from -0.0009 to $+0.0009$ with MC SEs $\approx$ 0.0004; not statistically distinguishable.	All values within $\pm$0.0007, MC SE = 0.0003; within error margin.	Ranges 0.0005–0.0009 with SE = 0.0004; not distinguishable.
% Gain in Precision	Up to 5.7% gain, but SE $\approx$ 4.7%; not reliable.	Max gain $\approx$ 3.3%, SE $\approx$ 4.5%; not meaningful.	Gains $<$ 1% with SE $\approx$ 4.5%; not interpretable.
Empirical SE	Small improvements (e.g., 0.0195 to 0.0190), MC SE = 0.0003; minimal impact.	Difference $\approx$ 0.0002 with MC SE = 0.0002; barely distinguishable.	All methods yield $\approx$ 0.0195; no practical difference.
Coverage	Increases from 92.95% to 96.60%, SEs 0.005; difference is statistically meaningful.	Improvement from 94.90% to 96.20%, SE 0.0047; marginally meaningful.	DCF methods reach 94.5%, TMLE at 92.95%; small but likely meaningful.
Relative % Error in SE	Ranges from -6.6% to +9.5%, SEs 1.5–1.7%; likely meaningful.	Ranges from -1.7% to +8.3%, SE 1.6%; moderate improvement.	Range: -6.6% to –0.8%, SE 1.6%; possible improvement trend.

D Real-world Analysis

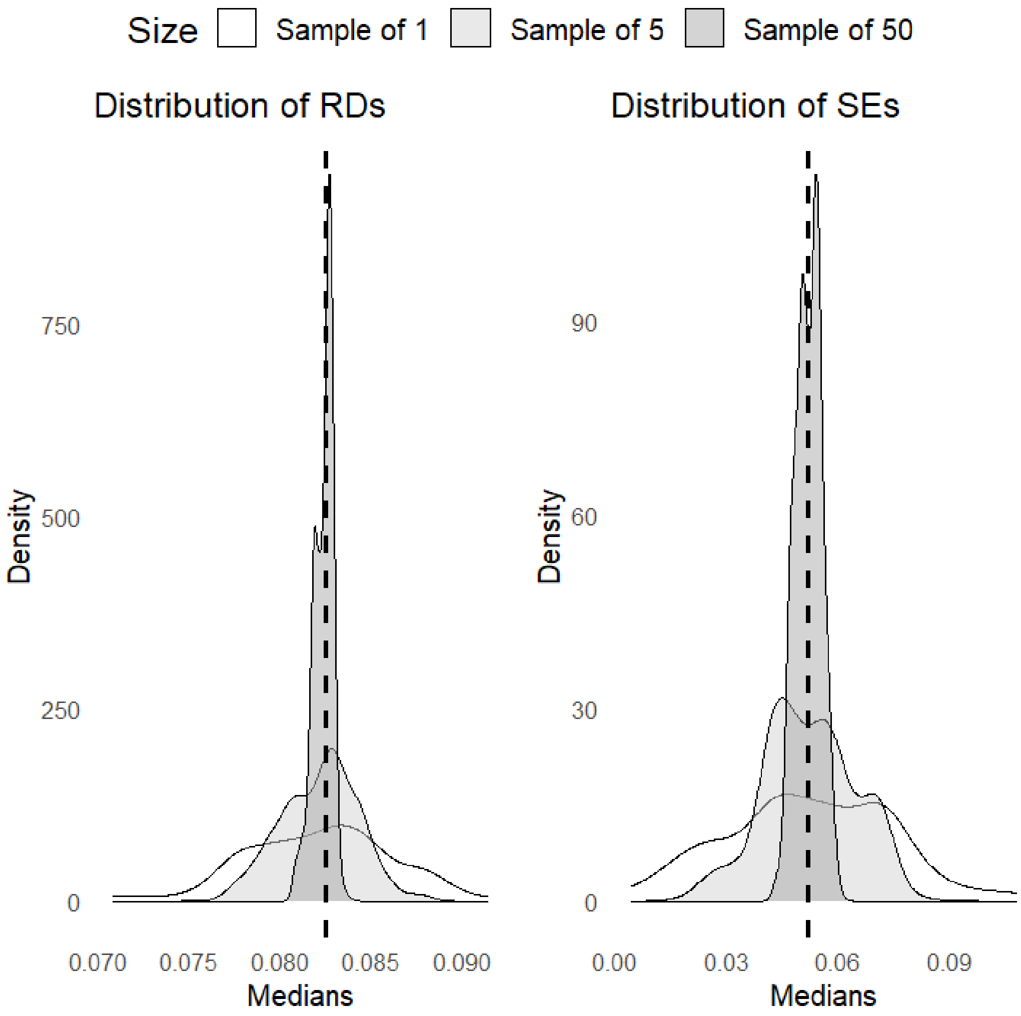

Appendix Figure D.1: Analyzing the association between obesity and diabetes risk using NHANES 2017-18 data, we generated 100 estimates with a 3-split repetition process with r=100 under Generalization 1. The distributions of overall risk differences (RDs) and standard errors (SEs) were generated by performing 10,000 iterations of random sampling (1, 5, and 50 estimates) without replacement. The dashed lines represent estimates of overall RD and SE from the 100 estimates.

E Future Directions

Variance calculation process: In line with prior work on DCF, we calculate variance by averaging the variances derived from separate splits of the dataset (Zivich and Breskin, 2021). It can be argued that this method may lead to a systematic overestimation of the true variance, especially as the number of splits increases. For instance, dividing a dataset into smaller subsets for sample splitting; each subset having fewer observations than the full dataset, typically results in higher variances for these subsets. Therefore, averaging these inflated variances might yield an overall variance estimate that exceeds what would be obtained using the entire dataset. This issue becomes increasingly notable in our simulations with more splits, as each split contains fewer data points, which in turn, inflates the variance estimate for each subset. Consequently, we observed that the model-based SE tends to increase with the number of splits. This highlights the necessity for further research to explore and compare alternative approaches to variance calculation in cross-fitting contexts, specifically examining their effects on the statistical

properties of the estimators involved.

Comparison with related methods: In the current work, we decided to focus exclusively on DCF, as its theoretical superiority over SCF was already demonstrated in earlier work (Newey and Robins, 2018). Future simulation studies could empirically compare these methods across a range of scenarios to provide clearer guidance on their respective advantages and applicability. While our simulation study provides valuable empirical evidence on the performance of the two generalizations of DCF TMLE, a rigorous theoretical analysis—including mathematical proofs of their asymptotic and finite-sample properties—remains an important direction for future research. Similarly, we did not expand on other related sample splitting procedures, such as cross-validation as previous research has already discussed extensively about those methods (Naimi et al., 2021; Balzer and Westling, 2021; Diaz, 2020).

Generally speaking, when choosing candidate learners for a super learner, a recent guideline suggested to consider the effective sample size, diversity of algorithms, computational feasibility, and appropriate screening methods to effectively tailor the learner to the data and predictive task (Phillips et al., 2023). Specifically, the guideline suggested selecting the number of cross-validation folds p based on the effective sample size (n_e), recommending higher p for smaller n_e and lower p for larger n_e for binary outcomes to balance bias, variance, and computational feasibility. Specifically, for $1,000 < n_e \leq 10,000$, their guideline suggests p close to 5 (also see 'tmle' package default automated choices for 'V.g' and 'V.Q'), which aligns well with our choice of p. Regarding learner selection, they emphasize the importance of incorporating both low-complexity (e.g., generalized linear models) and high-complexity (e.g., tree-based, ensemble) models. Our super learner library adheres to this principle, including logistic regression, generalized additive models, neural networks, and random forests, ensuring flexibility while maintaining interpretability. For the performance metric, they recommend selecting a loss function aligned with the study objective. Our study followed the negative log-likelihood loss, which are commonly used in causal inference applications. However, we acknowledge that alternative selection criteria could be explored in future work. Further research is needed to determine whether these general recommendations need to be specifically adapted for DCF implementations.

Appendix References

Sivaraman Balakrishnan, Larry Wasserman, and Min Xu. The fundamental limits of structure-agnostic functional estimation. *arXiv preprint arXiv:2305.04116*, 2023. URL https://arxiv.org/abs/2305.04116.

LB Balzer and T Westling. Demystifying statistical inference when using machine learning in causal research. *American Journal of Epidemiology*, 2021. doi: 10.1093/aje/kwab200.

V Chernozhukov, D Chetverikov, M Demirer, E Duflo, C Hansen, W Newey, and J Robins. Double/debiased machine learning for treatment and structural parameters. *The Econometrics Journal*, 21:1–68, 2018. doi: 10.1111/ectj.12097.

I Diaz. Machine learning in the estimation of causal effects: Targeted minimum loss-based estimation and double/debiased machine learning. *Biostatistics*, 21(2):353–358, 2020.

Evarist Giné and Richard Nickl. A simple adaptive estimator of the integrated square of a density. *Bernoulli*, 14 (1):47–61, 2008. doi: 10.3150/07-BEJ101. URL https://projecteuclid.org/euclid.bj/1201012100.

MH Mondol and ME Karim. Crossfit: An R Package to Apply Double Cross-fit Approach to TMLE in Causal Inference. *GitHub Repository*, 2023. https://github.com/momenulhaque/Crossfit.

MH Mondol and ME Karim. Towards robust causal inference in epidemiological research: Employing double cross-fit tmle in right heart catheterization data. *American Journal of Epidemiology*, page kwae447, 2024.

AI Naimi, Alan E Mishler, and EH Kennedy. Challenges in obtaining valid causal effect estimates with machine learning algorithms. *American Journal of Epidemiology*, 2021. doi: 10.1093/aje/kwab201.

Whitney K. Newey, Fushing Hsieh, and James Robins. Undersmoothing and bias corrected functional estimation. *Unpublished Manuscript*, 1998.

WK Newey and JR Robins. Cross-fitting and fast remainder rates for semiparametric estimation. *arXiv preprint arXiv:1801.09138*, 2018.

Rachael V Phillips, Mark J Van Der Laan, Hana Lee, and Susan Gruber. Practical considerations for specifying a super learner. *International Journal of Epidemiology*, 52(4):1276–1285, 2023.

James Robins, Lingling Li, Eric Tchetgen Tchetgen, and Aad van der Vaart. Higher order influence functions and minimax estimation of nonlinear functionals. *Institute of Mathematical Statistics Collections*, 3:335–421, 2008. doi: 10.1214/193940307000000527. URL https://projecteuclid.org/euclid.imsc/1207580738.

Aad W. van der Vaart and Jon A. Wellner. *Weak Convergence and Empirical Processes: With Applications to Statistics*. Springer Series in Statistics. Springer, New York, 1996. ISBN 978-0-387-94640-4.

Wenjing Zheng and Mark J. van der Laan. Asymptotic theory for cross-validated targeted maximum likelihood estimation. *U.C. Berkeley Division of Biostatistics Working Paper Series*, (Working Paper 266), 2010. URL https://biostats.bepress.com/ucbbiostat/paper266/.

PN Zivich and A Breskin. Machine learning for causal inference: on the use of cross-fit estimators. *Epidemiology*, 32(3):393–401, 2021. doi: 10.1097/EDE.0000000000001332.
